# Sub-6 GHz MIMO antenna system with enhanced performance based on compact decoupled PIFA pairs for future handheld devices

**DOI:** 10.1038/s41598-026-49235-2

**Published:** 2026-04-25

**Authors:** H. J. Basherlou, N. O. Parchin, C. H. See, A. S. I. Amar, S. A. Khaleel, M. Shen, M. S. Soliman

**Affiliations:** 1 School of Computing, Engineering & the Built Environment, Edinburgh Napier University, Edinburgh, UK; 2 Egyptian Technical Research and Development Centre, Cairo, 11618 Egypt; 3 Arab Academy for Science, Technology and Maritime Transport, Aswan, 81511 Egypt; 4 Department of Electronic Systems, Aalborg University, Aalborg, Denmark; 5 Department of Electrical Engineering, College of Engineering, Taif University, 21944 Taif, Saudi Arabia; 6 Research Center of Basic Sciences, Engineering and High Altitude, Taif University, Taif, Saudi Arabia

**Keywords:** 5G/6G, Wideband communications, Cellular applications, MIMO antennas, PIFA resonators, Aerospace engineering, Electrical and electronic engineering

## Abstract

This study presents a new MIMO antenna design with enhanced bandwidth and minimized mutual coupling, tailored for sub-6 GHz cellular applications. The design consists of eight compact planar inverted-F antennas (PIFAs) with discrete feeding, strategically arranged along the left and right edges of a 75 mm × 150 mm² smartphone PCB using an FR4 substrate. To ensure seamless integration with smartphone circuitry, a single-sided layout is adopted, where both the radiating elements and the ground-connected parasitic structures are printed on the same layer of the substrate. To improve bandwidth and isolation between closely spaced elements (edge-to-edge spacing = 1.5 mm), modified T-shaped stubs are placed between adjacent antennas. The design achieves wideband operation from 3.25 to 4.25 GHz with good impedance matching (S₁₁ < − 10 dB), mutual coupling suppression (~ 13 dB), and excellent diversity metrics such as low envelope correlation coefficient (ECC < 0.05) and total active reflection coefficient (TARC < − 20 dB). The antenna performance remains robust under practical use cases including talk-mode and double-hand scenarios. It also supports a full-ground configuration by repositioning elements along the periphery, with negligible degradation in matching. Further, the link budget analysis confirms reliable communication over long distances (> 1.5 km), accounting for polarization and impedance mismatch losses. Finally, to demonstrate scalability, a broadband mmWave phased array (27–44 GHz) is co-designed on the same PCB, enabling future sub-6 GHz and mmWave 5G/6G smartphone integration. With its compact, wideband, low-SAR and multi-mode operation, the proposed antenna system is a strong candidate for next-generation handheld wireless platforms.

## Introduction

The rapid advancement of wireless communication technologies has driven an unprecedented demand for higher data rates, lower latency, and improved service quality. The growing reliance on high-speed connectivity for mobile broadband and the Internet of Things (IoT) has necessitated the development of next-generation wireless standards^1^. In response, 5G has emerged as a transformative paradigm, addressing the limitations of 4G LTE by offering significantly enhanced capacity, ultra-low latency, and more reliable connectivity. Unlike its predecessors, 5G is designed to support a broad range of services such as enhanced mobile broadband (eMBB). These capabilities position 5G and its future evolution into 6G as essential enablers for emerging applications across diverse domains. A core technology underpinning the success of both 5G and anticipated 6G networks is Multiple-Input Multiple-Output (MIMO). MIMO improves spectral efficiency and overall capacity by employing multiple antennas to exploit spatial diversity and multipath propagation^2^. This not only enhances data throughput but also strengthens link robustness, ensuring more reliable wireless communication in dynamic environments. Traditional MIMO systems, such as those used in 4G LTE, typically utilize configurations like 2 × 2 or 4 × 4. However, to meet the demanding performance targets of 5G and beyond, massive MIMO architectures have emerged, incorporating significantly larger arrays with often 8, 16, or higher number of elements. In addition to increasing capacity, MIMO leverages advanced signal processing techniques such as spatial multiplexing, beam-forming, and diversity gain to further optimize network performance^3,4^.

The integration of MIMO technology in 5G handheld devices is particularly challenging due to the constraints of compact device form factors, limited PCB space, and the necessity for multi-band operation. A major design consideration is the development of wideband and efficient antenna systems that can support broad and multiple frequency bands while maintaining a compact footprint. Furthermore, mutual coupling between adjacent antenna elements can negatively impact system performance by increasing correlation between signals, leading to reduced diversity gain and lower overall efficiency. To address these challenges, sophisticated antenna engineering techniques, such as decoupling structures, electromagnetic bandgap (EBG) materials, and metamaterials, have been explored to enhance isolation and improve bandwidth performance^5^. Printed antennas have gained considerable attention for their ability to provide compact, low-profile, and cost-effective solutions for smartphone integration^6^. Among various printed antenna structures, Planar Inverted-F Antennas (PIFAs) are extensively used in mobile devices due to their favorable characteristics, including compact size, low profile, and good impedance matching. PIFAs can be easily integrated with smartphone circuitry, and their design can be optimized to achieve the required bandwidth and isolation performance. However, traditional PIFA-based MIMO configurations often suffer from narrow bandwidth and high mutual coupling, necessitating innovative modifications to enhance performance^7^.

Significant research efforts have focused recently on advancing and developing MIMO antenna systems suitable for sub-6 GHz cellular networks, focusing on improving impedance bandwidth, isolation, and radiation characteristics^8–25^. However, many existing designs exhibit limitations, such as bulky structures, inefficient utilization of PCB space, and performance degradation due to high coupling effects. To overcome these constraints, this study proposes a new eight-element modified PIFA MIMO antenna. The proposed antenna configuration is specifically designed to achieve broad impedance bandwidth and low mutual coupling while maintaining a compact planar form factor suitable for smartphone platforms. The antenna elements are strategically positioned along the left and right edges of the smartphone mainboard, resulting in an optimized 75 × 150 mm^2^ footprint on an FR4 substrate. To further enhance isolation and bandwidth performance, modified T-shaped decoupling strips are introduced between adjacent PIFA resonators. These structures suppress surface-wave propagation and reduce undesired electromagnetic coupling, thereby improving the overall efficiency and stability of the radiators. The proposed antenna system operates across the 3.25–4.25 GHz band, covering a significant portion of the sub-6 GHz spectrum used for modern 5G communication systems. The antenna performance is extensively evaluated using CST simulations^26^, focusing on key parameters such as S-parameters, radiation characteristics, and MIMO diversity performance. By combining efficient spatial arrangement with advanced decoupling techniques, the proposed compact wideband antenna system effectively addresses key challenges associated with smartphone MIMO antenna integration. In addition to the compact edge-mounted configuration, the antenna system demonstrates robust performance under realistic user-interaction conditions, including talk-mode and double-hand operation, while maintaining SAR compliance even under simultaneous multi-port excitation. Unlike conventional sub-6 GHz smartphone MIMO antennas, the proposed design uniquely combines a single-sided PCB implementation with closely spaced PIFA pairs and modified T-shaped decoupling structures. This approach enables simultaneous achievement of wideband operation, compact layout, and reduced mutual coupling without requiring multilayer structures or complex decoupling networks. In addition, the inclusion of system-level evaluations such as SAR analysis, user-interaction scenarios, and mmWave scalability further distinguishes the proposed work from existing designs, providing a practical and forward-compatible solution for next-generation handheld devices. To further demonstrate the scalability of the proposed platform, a compact proof-of-concept mmWave phased-array antenna operating from 27 to 44 GHz is also developed and analyzed for potential co-integration on the same smartphone PCB. The phased array exhibits wideband response and beam-steering capability, illustrating the feasibility of combining sub-6 GHz MIMO systems with directional mmWave communication modules for future multi-band 5G and beyond wireless devices^27^. The main contributions of this work can be summarized as follows:

1. A compact eight-element PIFA-based MIMO antenna architecture composed of closely spaced antenna pairs is proposed for sub-6 GHz smartphone applications.
2. T-shaped decoupling structures are introduced between adjacent PIFA elements to simultaneously enhance impedance bandwidth and suppress mutual coupling.
3. The antenna system demonstrates robust performance under realistic user-interaction conditions, including talk-mode and double-hand scenarios, while maintaining safe SAR levels and excellent MIMO diversity characteristics.
4. The design supports single-sided PCB implementation and full-ground smartphone configurations, enabling simplified fabrication and practical device integration.
5. The work demonstrates the potential coexistence and integration of a broadband mmWave phased-array antenna on the same platform, enabling scalable architectures capable of supporting both sub-6 GHz MIMO communication and directional mmWave beam-steering systems for future 5G and beyond networks.

## The suggested antenna configuration and its key properties

**Fig. 1:**
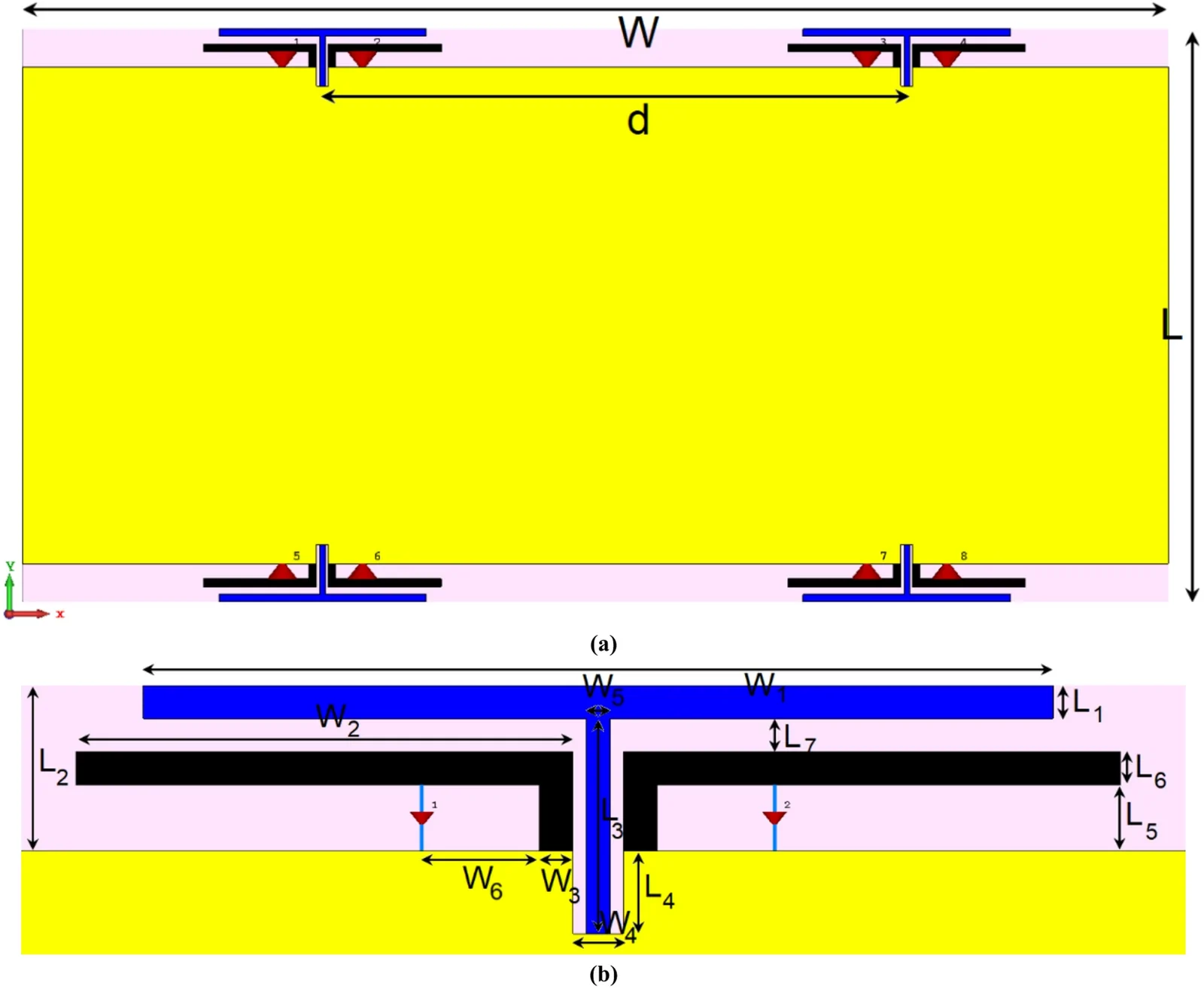
(**a**) Schematic of the suggested MIMO, (**b**) the design details of its pairs.

Figure 1 illustrates the schematic of the suggested MIMO antenna. As shown, its layout is straightforward and efficient. The design features overall dimensions of 75 × 150 mm² and integrates an array of 2 × 4 modified PIFA resonators, including four pairs with each pair complemented by T-shaped stubs. The dimensions of the antenna parameters are detailed in Table 1. To excite the antenna elements, each PIFA radiator is fed using a 50-Ω excitation configuration. In the electromagnetic simulations, discrete ports are assigned between the feed point and the ground reference. For the fabricated prototype, the antenna elements are excited through coaxial SMA connectors, where the inner conductor of the coaxial cable is soldered to the feed point of the PIFA radiator while the outer conductor is connected to the ground plane of the smartphone PCB. In addition, a grounded shorting strip connects the radiating arm to the ground plane, forming the classical PIFA structure that supports quarter-wavelength (λ/4) resonance and ensures efficient power transfer from the RF source to the antenna element. As shown in Fig. 1 and detailed in Table 1, the adjacent PIFAs within each pair are placed at a very close edge-to-edge distance of only W_4_ = 1.5 mm and port-to-port distance of 10 mm. This value represents a narrow physical gap, well within the range of strong electromagnetic coupling, and is precisely why a decoupling structure (the T-shaped structures) is required between them. The larger distance d = 76.5 mm refers to the spacing between the centers of separate PIFA pairs, which is intentionally larger to ensure pattern diversity and spatial separation in the MIMO array layout.

**Table 1:** Design values of the antenna parameters.

Parameter	W	L	W_1_	L_1_	W_2_	L_2_	W_3_	L_3_
Value (mm)	150	75	27	1	14.8	5	1	6.5
Parameter	W_4_	L_4_	W_5_	L_5_	W_6_	L_6_	L_7_	d
Value (mm)	1.5	2.5	27.1	2	3.5	1	1	76.5

**Fig. 2:**
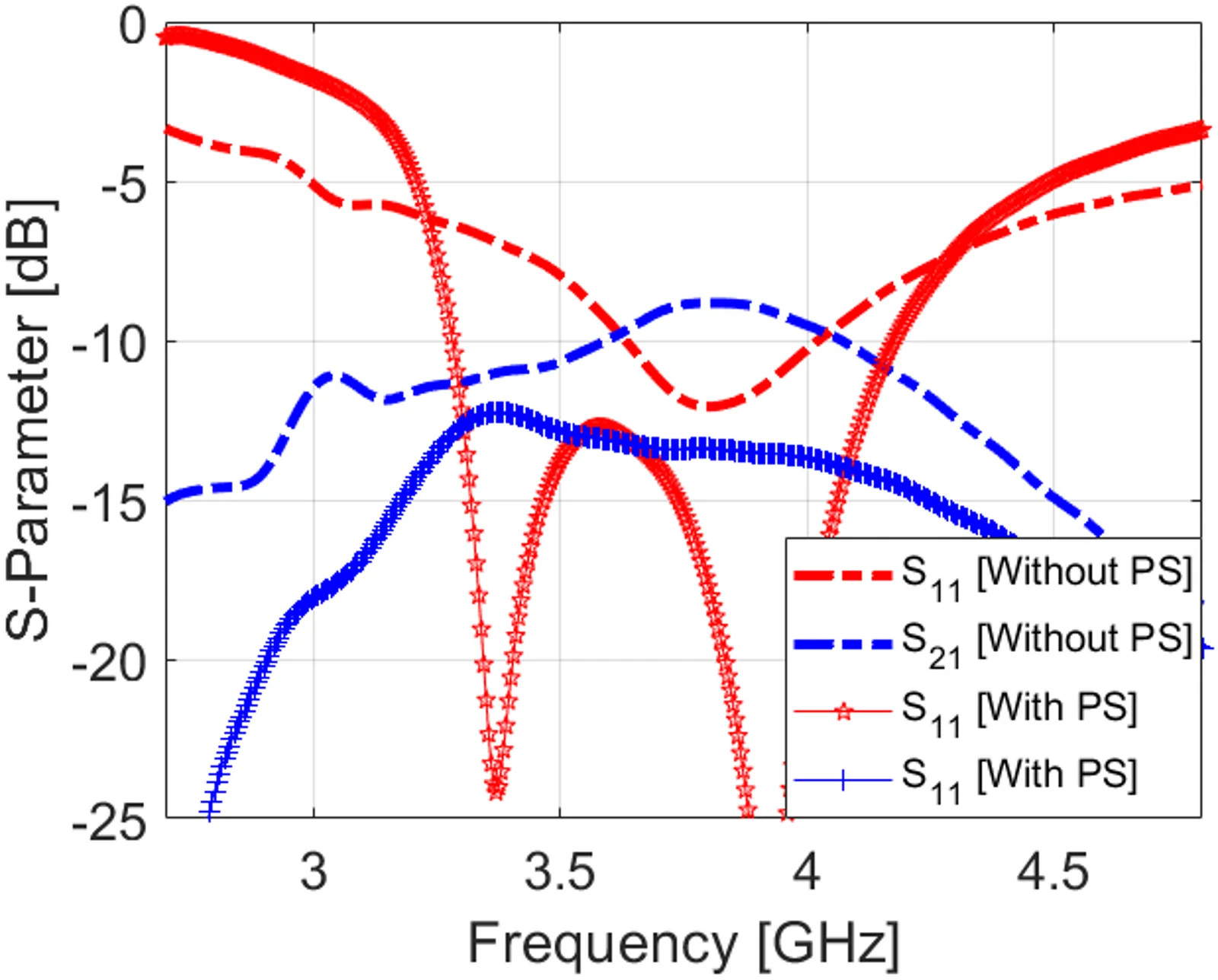
S_11_/S_21_ results with and without the T-shaped strip between the PIFAs.

The use of these modified resonators offers multiple benefits. Firstly, it improves the antenna’s frequency response and matching performance. Secondly, it ensures symmetrical radiation effectively covers both the top and bottom regions. Additionally, PIFAs are known for their inherently low profile, robust performance, and versatility in supporting wideband and multiband operations. These attributes make them well-suited for sub-6 GHz applications, where compactness and reliability are crucial. This design is optimized to deliver reliable and efficient performance for smartphone applications, balancing a compact form factor with desirable radiation characteristics. The proposed antenna configuration was developed following a systematic design procedure. Initially, the fundamental dimensions of the PIFA radiator were estimated using the classical quarter-wavelength resonance model, which relates the effective electrical length of the radiator to the operating frequency. The initial geometry was selected to place the primary resonance within the targeted sub-6 GHz band. Subsequently, a T-shaped parasitic strip was introduced between adjacent PIFA elements to provide an additional current path and reduce electromagnetic coupling between closely spaced antennas. This parasitic element also contributes to the generation of a secondary resonance, leading to bandwidth enhancement. Finally, key geometrical parameters such as the PIFA arm length, feed position, and parasitic strip dimensions were optimized through parametric simulations to achieve improved impedance matching, wider bandwidth, and enhanced isolation suitable for smartphone MIMO applications. Figure 2 shows the simulated scattering (S) parameters of the antenna pair for configurations with and without the T-shaped parasitic element. The comparison clearly indicates that the presence of the T-shaped structure leads to a substantial expansion of the impedance bandwidth, thereby improving the overall frequency response of the antenna system. Additionally, the strip effectively reduces mutual coupling between the PIFA pairs, improving isolation and ensuring more efficient functionality of the closely spaced elements. This improvement underscores the critical role of the T-shaped strip in improving the antenna’s behavior for modern wireless applications. As illustrated, the reflection coefficients (S_11_) of the antenna elements improve from a narrow bandwidth of 3.6–4 GHz to a wider operational range of 3.25–4.25 GHz, resonating specifically at 3.4 GHz and 3.9 GHz. This reflects optimal impedance compatibility, leading to low reflection losses and effective energy transfer. Furthermore, the mutual coupling between adjacent PIFAs is significantly reduced, with isolation levels (S_21_) improving from − 8 dB to better than − 13 dB, as shown in the results. This enhanced isolation effectively mitigates interference between antenna elements, contributing to improved MIMO performance^28^.

The resonance behavior of the proposed PIFA-based antenna structure is governed by the classical λ/4 model, which is characteristic of grounded PIFA configurations. In such structures, the current travels from the feed point to an open-ended radiating element, forming a quarter-wavelength standing wave. This model provides an accurate prediction of the fundamental resonance frequency based on the effective electrical length and dielectric loading, as described by:

1 $$\:{f}_{r}=\frac{c}{4{L}_{eff}\sqrt{{\epsilon\:}_{eff}}}$$

Considering the effective dielectric constant of ε_eff_ ≈ 4.3 for the FR4 substrate, and based on the antenna’s physical layout, the effective electrical length associated with the 3.75 GHz resonance is L_eff_ = W_2_ + 2L_5_ + 0.5L_6_ = 14.8 + 4 + 0.5 = 19.3 mm

$$\:{f}_{r}=\frac{3\times\:{10}^{8}}{4\times\:0.0193\sqrt{4.3}}\approx\:3.75$$.

To understand the dual-resonance behavior observed in the simulated and measured results, the effective path lengths corresponding to the two operating frequencies are also calculated as follow:

$$\:{L}_{eff,3.4\:GHz}=\frac{3\times\:{10}^{8}}{4\times\:3.4\times\:{10}^{9}\sqrt{4.3}}\approx\:21.29\:mm\approx\:{0.5W}_{1}+{L}_{1}+{L}_{3}$$

$$\:{L}_{eff,3.9\:GHz}=\frac{3\times\:{10}^{8}}{4\times\:3.9\times\:{10}^{9}\sqrt{4.3}}\approx\:18.56\:mm\approx\:{W}_{2}+{W}_{3}+{L}_{4}+{L}_{5}$$

**Fig. 3:**
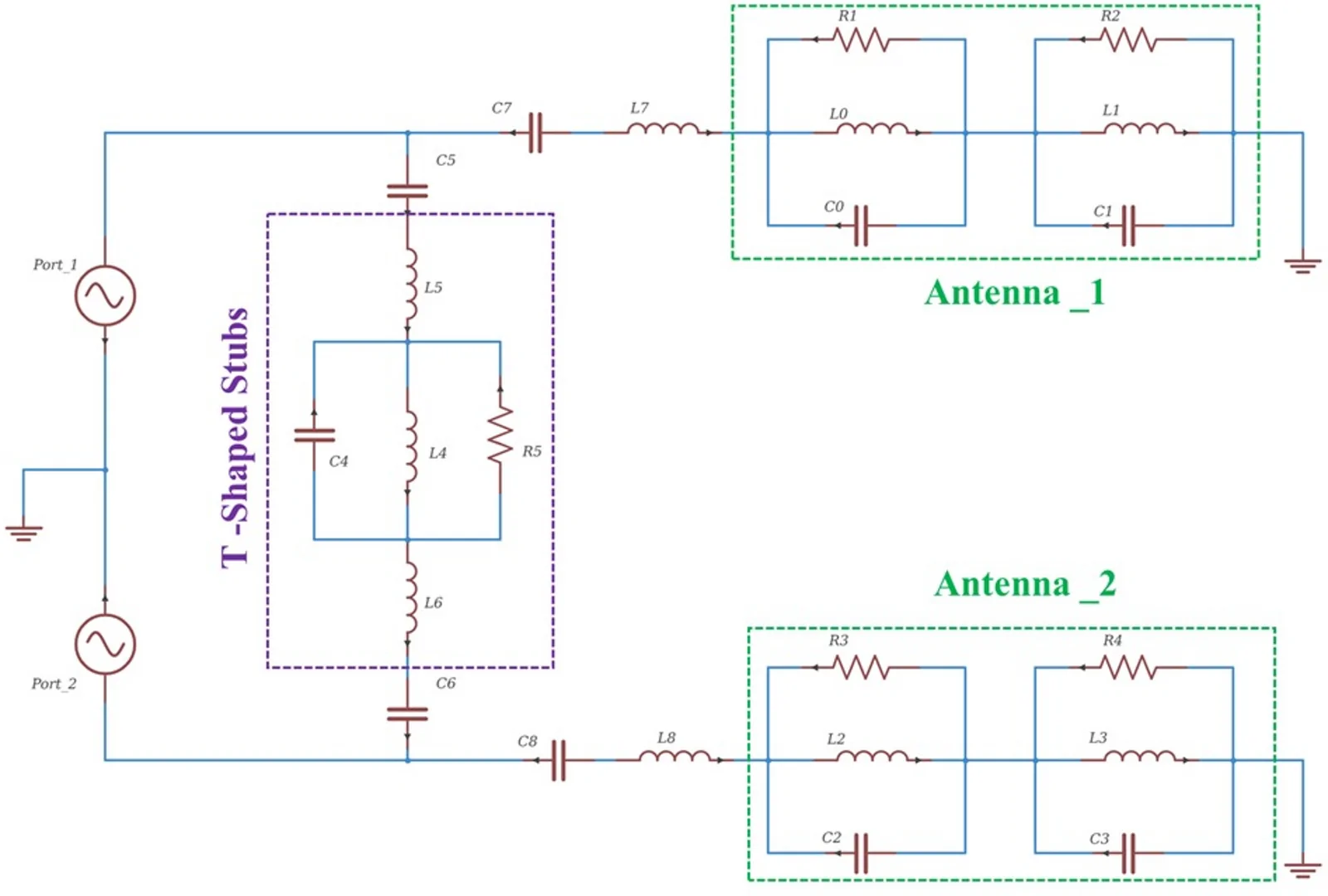
The equivalent circuit of its pairs.

These values support the interpretation that the lower resonance (3.4 GHz) originates from the longer current path introduced by the T-shaped parasitic structure, while the higher resonance (3.9 GHz) corresponds to the more compact PIFA arm. To better understand the resonance behavior and decoupling mechanism of the proposed antenna system, an equivalent circuit model of a representative antenna pair is illustrated in Fig. 3. Each PIFA radiator is modeled using a series RLC branch (e.g., $\:{R}_{1},{L}_{0},{C}_{0}$) to capture its resonant behavior. The T-shaped decoupling structure between the two antennas is represented by a parallel LC tank circuit ($\:{L}_{4},{C}_{4}$) in series with additional parasitic elements to model its frequency-selective isolation properties. The coupling network also includes matching and feed components, represented by additional lumped inductors and capacitors ($\:{L}_{5}$–$\:{L}_{8}$, $\:{C}_{5}$–$\:{C}_{8}$) on both paths to simulate transmission-line effects and inter-element coupling. The actual component values of the equivalent model, optimized to replicate the measured $\:{S}_{11}$and $\:{S}_{21}$responses, are summarized in Table 2. Since the full MIMO system comprises four identical antenna pairs, this model can be extended to represent the full 8-element array by replicating the pair-wise configuration and including second-order mutual coupling between adjacent pairs. This equivalent circuit offers valuable insight into the dual-resonance tuning and isolation mechanism enabled by the T-shaped structure.

**Table 2:** Actual values of the equivalent circuit parameters.

Parameter	R1	R2	R3	R4	R5	C0
Value	88.3 Ω	50 Ω	120 Ω	150 Ω	80 Ω	1.61 fF
Parameter	C1	C2	C3	C4	C5	C6
Value	40.2 fF	2.12 fF	7.52 fF	9.84 fF	3.5 fF	9.1 fF
Parameter	C7	C8	L0	L1	L2	L3
Value	1.25 fF	3.75 fF	0.71 nH	0.61 nH	0.47 nH	0.56 nH
Parameter	L4	L5	L6	L7	L8	
Value	0.131 nH	18.6 nH	50 nH	0.25 nH	0.75 nH	

**Fig. 4:**
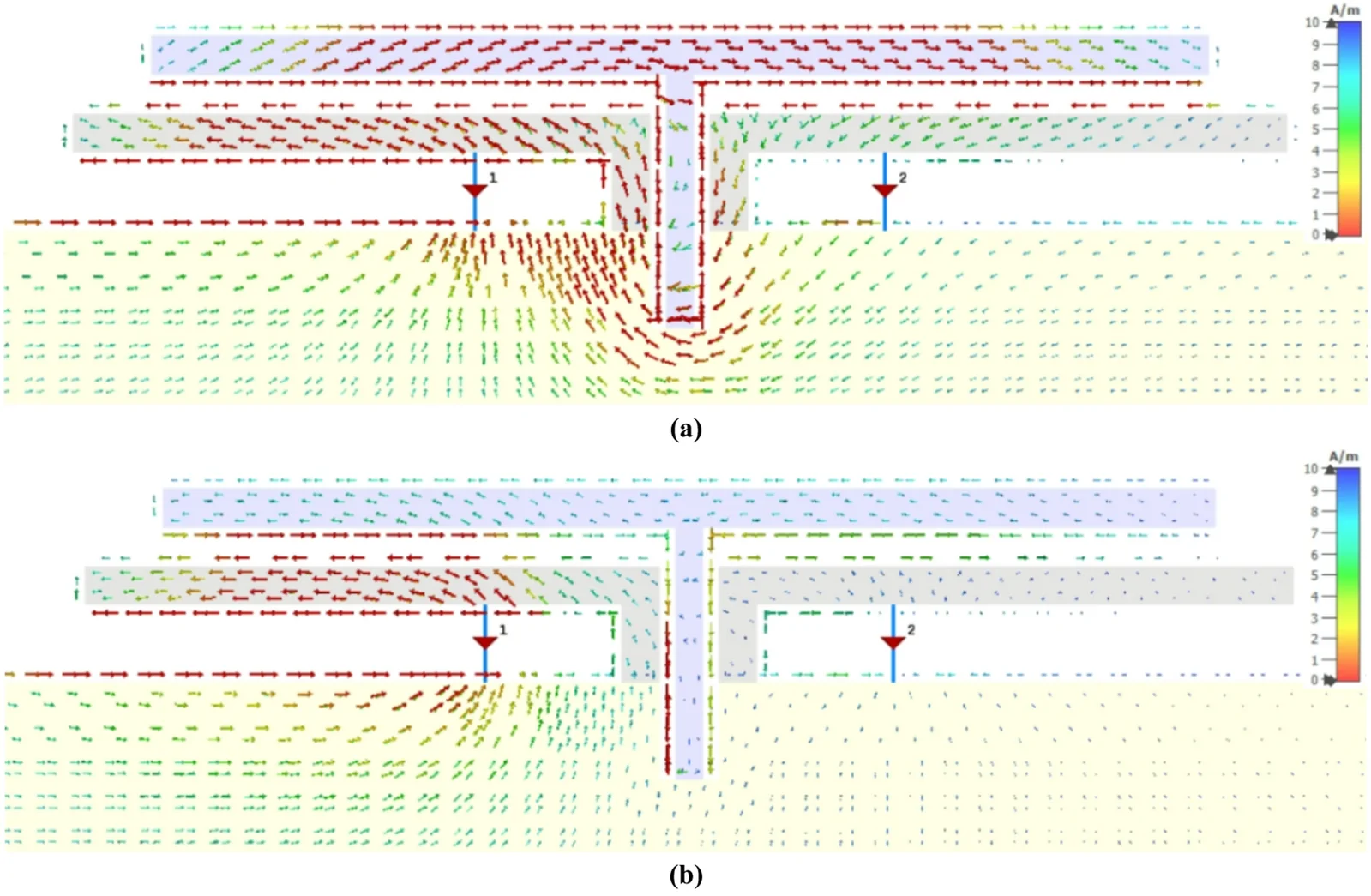
Surface current densities of a PIFA at (**a**) 3.4 GHz and (**b**) 3.9 GHz.

To analyze and validate the enhanced characteristics of the closely spaced PIFA elements, the current distributions at 3.4 and 3.9 GHz for port 1 are shown in Fig. 4. At the lower resonant frequency of 3.4 GHz, strong surface currents are observed along the T-shaped parasitic element, which forms the extended current path of the structure, confirming excitation of a quarter-wavelength (λ/4) resonant mode. Conversely, at 3.9 GHz, the current distribution is mainly confined to the shorter PIFA radiator, indicating that this element dominates radiation at the higher resonance. This behavior highlights the advantage of incorporating the T-shaped strip between the resonators. The strip introduces an additional resonance that not only broadens the operational bandwidth but also mitigates mutual coupling between the PIFAs. Furthermore, the analysis emphasizes the inverse relationship between antenna size and operating frequency, wherein smaller dimensions correspond to higher resonant frequencies. This behavior directly confirms the theoretical λ/4 path-length analysis, where the longer path (~ 21.3 mm) at 3.4 GHz results from the inclusion of the T-shaped parasitic structure, while the shorter path (~ 18.6 mm) at 3.9 GHz is confined to the main PIFA radiator. Moreover, the reduction in mutual coupling between closely spaced PIFA elements is primarily achieved through the introduction of the T-shaped parasitic stub, which modifies the surface current distribution and electromagnetic field interaction between adjacent elements. As observed in the surface current distributions, the parasitic structure introduces an alternative current path that redirects surface currents away from neighboring antennas, thereby reducing direct coupling. In addition, the T-shaped stub behaves as a resonant decoupling element that introduces a high impedance path at the operating frequencies, effectively suppressing current leakage between adjacent radiators^29^. This mechanism can also be interpreted as a combination of field cancellation and impedance transformation, where induced currents on the parasitic structure generate opposing electromagnetic fields that reduce mutual coupling. The current flow visualization therefore validates that the dual resonance behavior arises from mode splitting due to distinct current paths^30^.

The presence of two closely spaced resonances significantly improves impedance matching and expands the antenna’s operational bandwidth. This is described using the fractional bandwidth (FBW):

2 $$\:FBW=\frac{{f}_{H}-{f}_{L}}{{f}_{0}}\approx\:\frac{2}{Q}$$

From the S-parameter results shown in Fig. 2:

Without the T-shaped PS: 3.6–4.0 GHz → Δf = 0.4 GHz → FBW ≈ 10.5%.

With the T-shaped PS: 3.25–4.25 GHz → Δf = 1.0 GHz → FBW ≈ 26.7%.

This confirms that the T-shaped parasitic structure introduces a second, closely coupled resonance, broadening the impedance matching range and reducing the quality factor. In addition to bandwidth enhancement, the T-shaped parasitic strip also reduces mutual coupling between adjacent PIFA elements. This is achieved through field cancellation and impedance transformation. Electromagnetically, the PS modifies the mutual impedance as:

3 $$\:{Z}_{12}^{new}={Z}_{12}+{Z}_{PS}{=Z}_{12}+(j\omega\:L-\frac{1}{j\omega\:L})$$

**Fig. 5:**
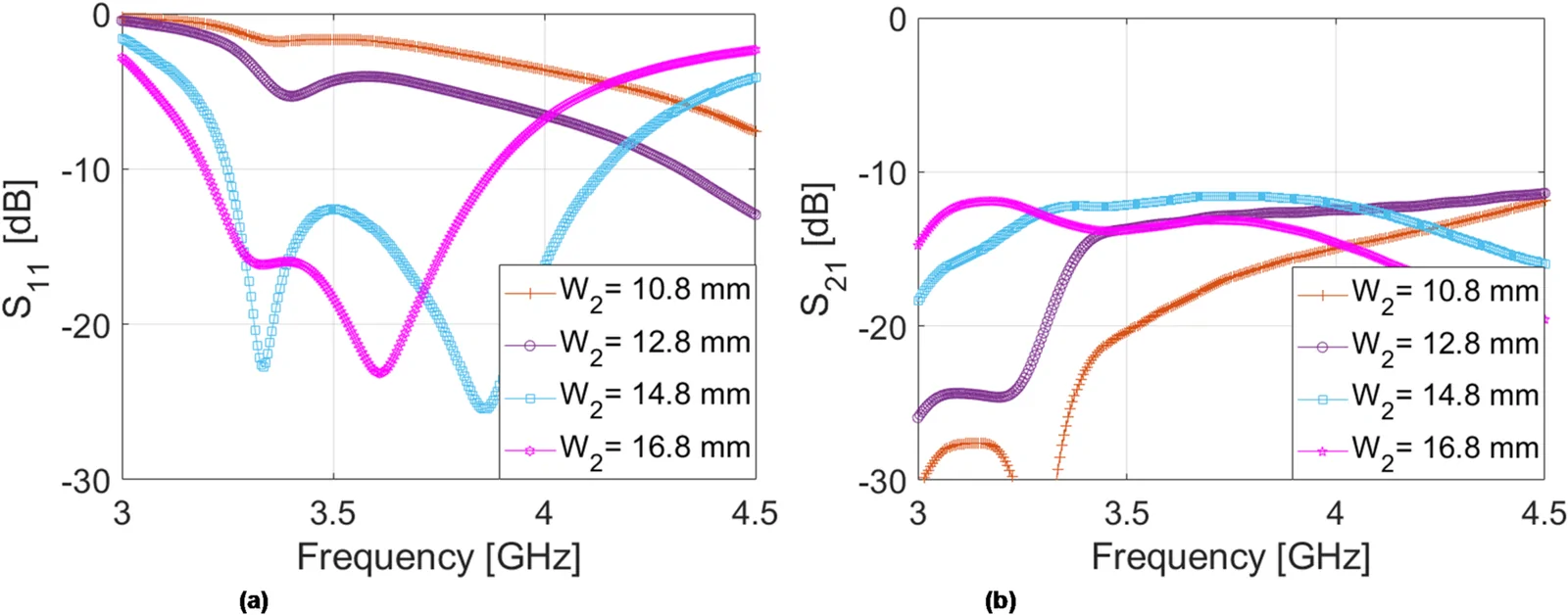
(**a**) S_11_ and (**b**) S_21_ parameters for various values of W_2_.

**Fig. 6:**
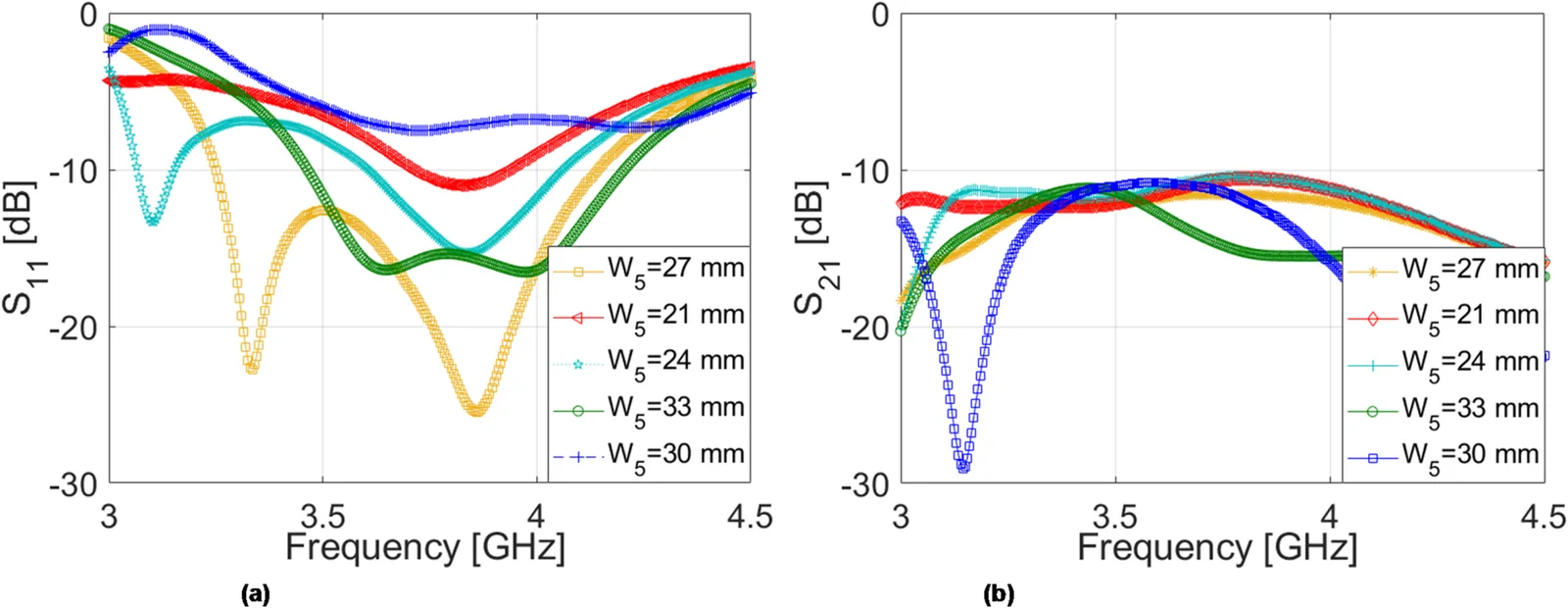
(**a**) S_11_ and (**b**) S_21_ parameters for various values of W_5_.

**Fig. 7:**
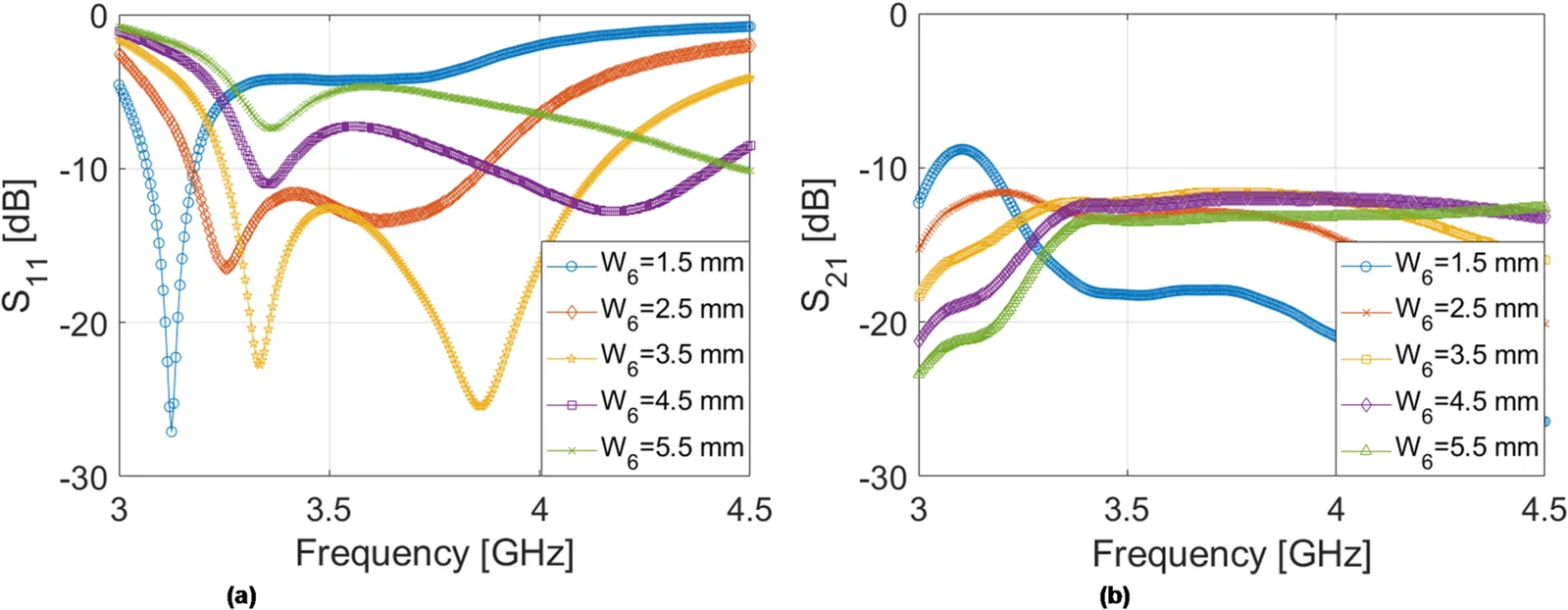
(**a**) S_11_ and (**b**) S_21_ parameters for various values of W_6_.

At its resonant frequency (~ 3.4 GHz), the PS presents high impedance, effectively suppressing current transfer between adjacent radiators. This effect is verified by the improvement in S_21_ isolation, increasing from − 8 dB (without PS) to better than − 13 dB (with PS), as seen in Fig. 2. This analysis confirms the essential role of the T-shaped parasitic strip in enabling dual-resonance operation, wideband impedance matching, and improved mutual isolation. The parametric studies presented in Figs. 5, 6 and 7 were conducted prior to the final design to optimize the antenna’s performance and achieve the best possible S-parameter characteristics. Specifically, the design parameters W_2_ (PIFA arm length), W_5_ (T-decoupler width), and W_6_ (feeding point) have been carefully analyzed. In each simulation, one parameter is varied while keeping all others constant, as detailed in Table 1. Figure 5 illustrates the S-parameter response (S_11_/S_21_) of the antenna when W_2_ is adjusted. The results indicate that changes in W_2_ influence impedance matching between the feeding ports. Notably, when W_2_ is set to 14.8 mm, the antenna exhibits optimal performance.

Another crucial parameter under investigation is the width of the T-shaped decoupling strip (W_5_). As depicted in Fig. 6, varying W_5_ between 21 mm and 33 mm produces different responses in the S_11_ and S_21_ characteristics. Among the tested values, W_5_ = 24 mm provides the widest bandwidth and the most favorable mutual coupling characteristics, making it the optimal choice. Additionally, the placement of the feeding point (W_6_) plays a significant role in shaping the antenna’s S-parameters, as it influences the shift in lower and higher bandwidth frequencies. Figure 7 demonstrates how varying W_6_ from 1.5 mm to 5.5 mm affects the performance. The results indicate that W_6_ = 3.5 mm yields the most suitable S-parameter characteristics, ensuring balanced impedance matching and minimal coupling effects. The wide impedance bandwidth (3.25–4.25 GHz) is achieved through the generation of dual resonant modes resulting from the combined effect of the PIFA radiator and the T-shaped parasitic structure. The lower resonance is associated with the extended current path introduced by the parasitic element, while the higher resonance is dominated by the main PIFA radiator. The interaction between these two resonances results in bandwidth merging, leading to an overall bandwidth enhancement. This behavior is further validated through the parametric studies presented in Figs. 5, 6 and 7, where variations in key geometrical parameters demonstrate their influence on resonance shifting and bandwidth merging^31^.

**Fig. 8:**
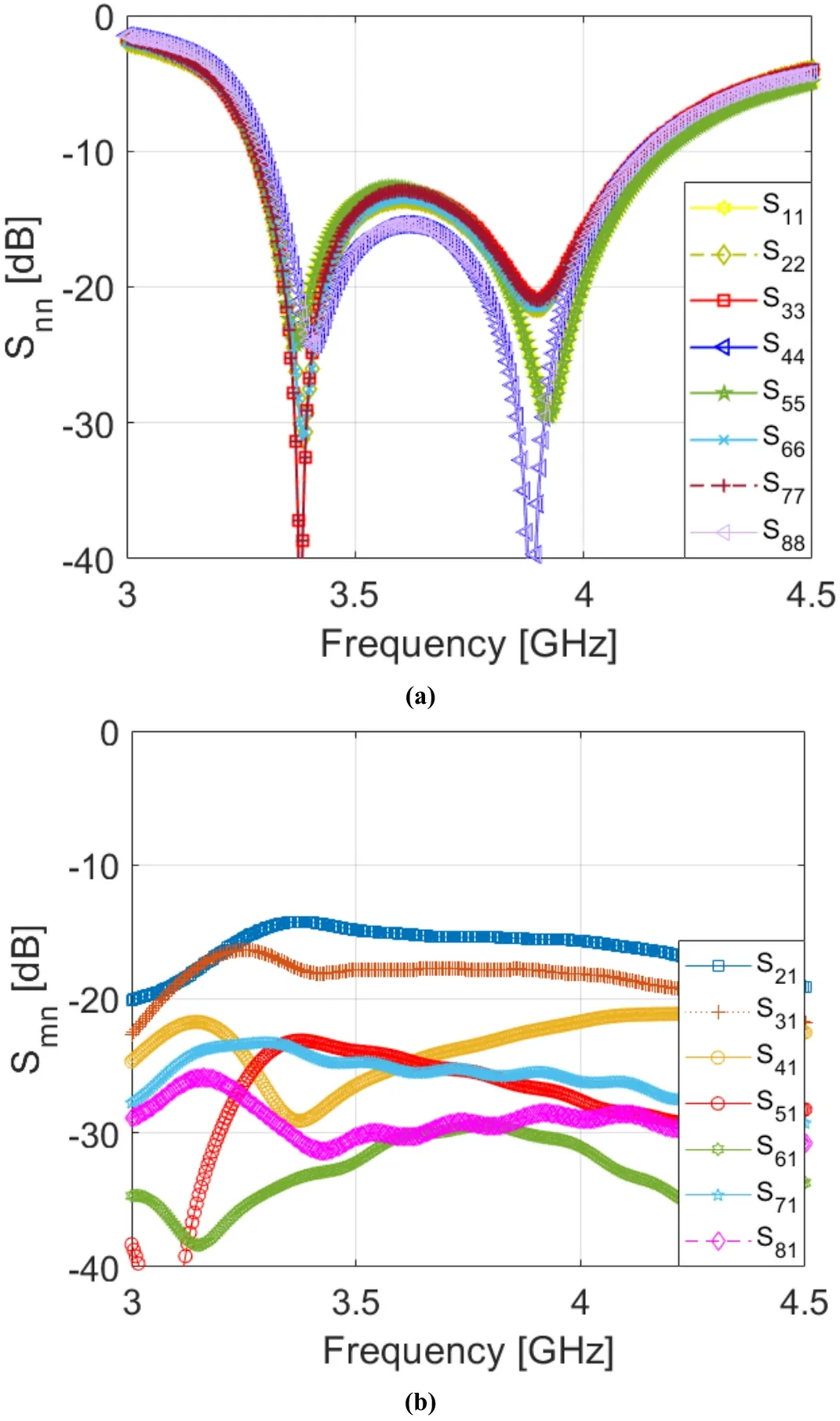
(**a**) S_nn_ and (**b**) S_nm_ results of the PIFA elements.

**Fig. 9:**
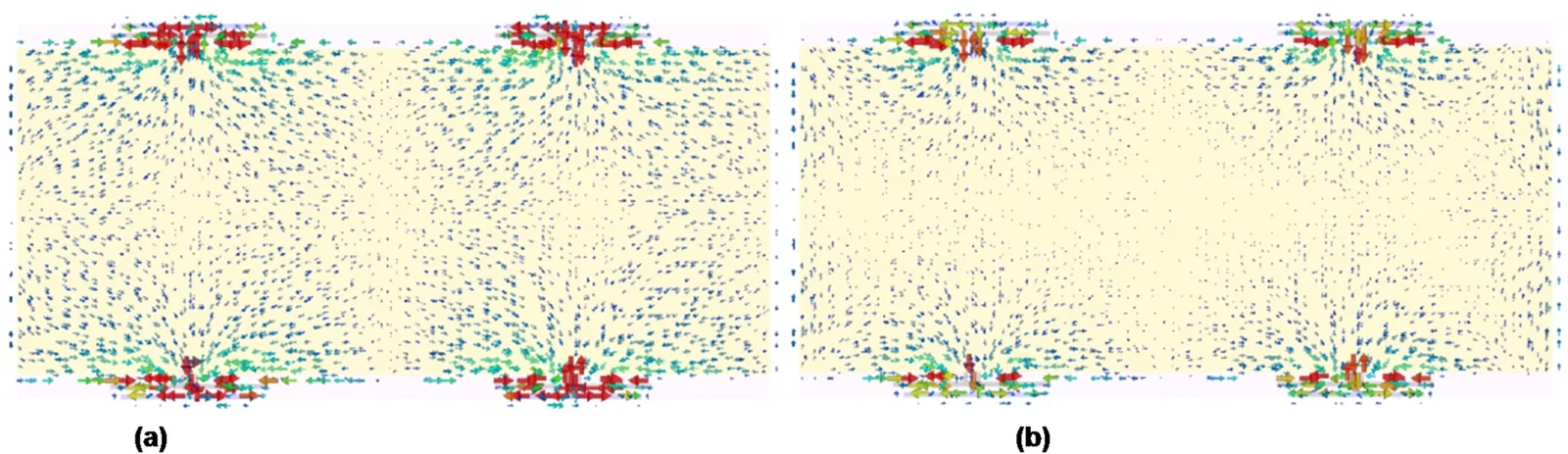
Surface current densities of the smartphone board at (**a**) 3.4 GHz and (**b**) 3.9 GHz.

Figure 8 depicts the S-parameters of the array, demonstrating stable and efficient performance throughout the designated frequency range. As shown in Fig. 8 (a), the reflection coefficients (S_nn_) for all antenna elements exhibit excellent matching, supporting a broad frequency band from 3.25 to 4.25 GHz. This highlights the effectiveness of the design in achieving a well-matched impedance profile over the desired spectrum. Furthermore, as shown in Fig. 8 (b), the coupling among the antenna elements is significantly reduced, with isolation levels (S_nm_) below − 13 dB. This indicates strong isolation between the elements, which is essential for minimizing interference and enhancing the array’s overall performance. To validate the electromagnetic performance of the proposed single-sided configuration where the radiating PIFA structures, the T-shaped stubs and the ground plane are etched on the same side of the substrate, a surface current distribution analysis was conducted at the two main resonant frequencies (3.4 GHz and 3.9 GHz). As shown in Fig. 9, the current density remains well-confined to the intended radiating elements and decoupling structures, with no abnormal spreading or disruption across the substrate. This indicates that the radiation mechanism is preserved and that the single-layer layout does not degrade antenna functionality^32^.

**Fig. 10:**
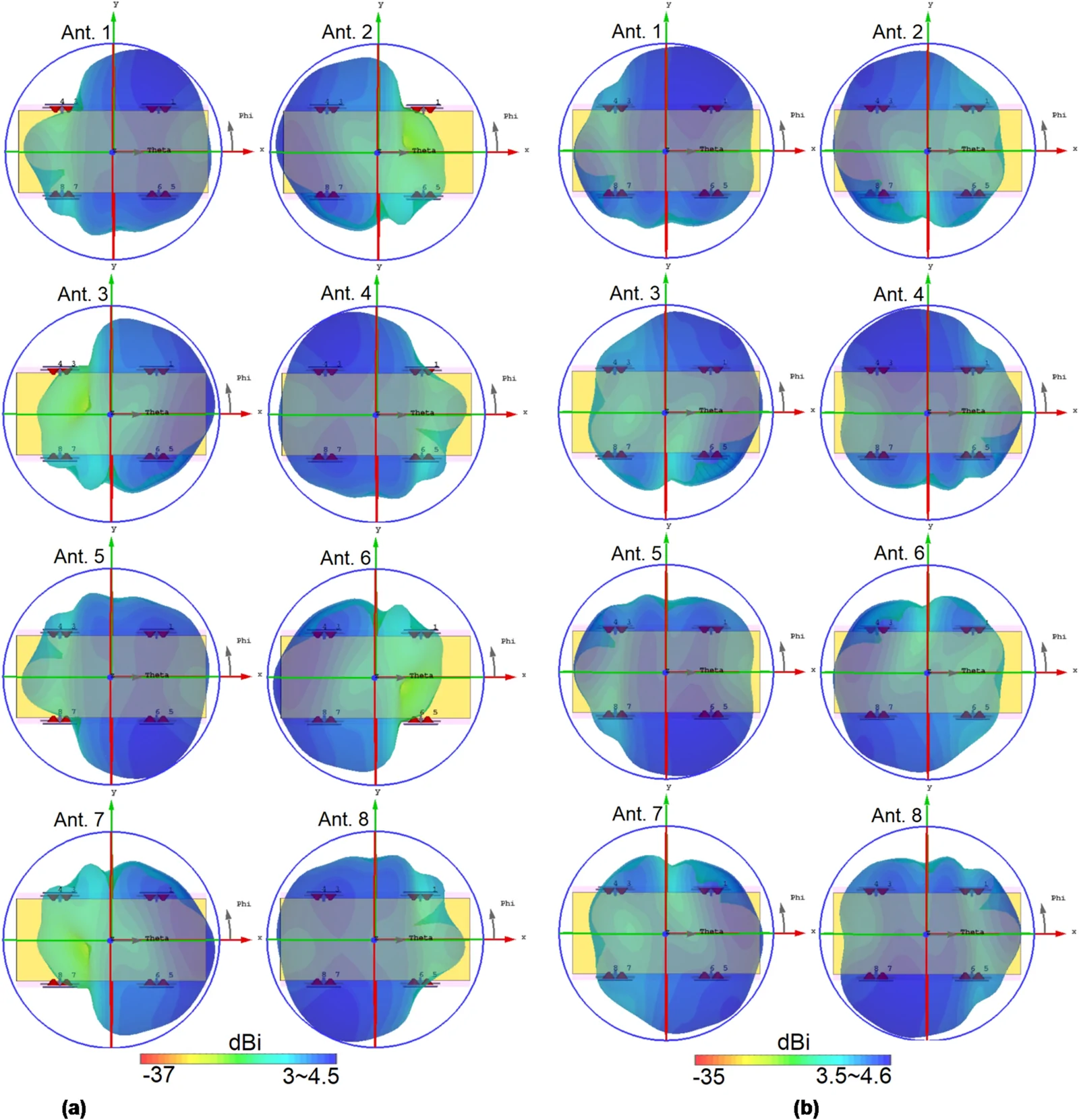
3D radiations at (**a**) 3.4 GHz and (**b**) 3.9 GHz.

Figure 10 illustrates the 3D radiation of the antenna elements, along with respective gain levels at 3.4 GHz and 3.9 GHz. As depicted, the 8-antenna configuration delivers well-structured radiation characteristics. Additionally, the observed gain levels are sufficient to ensure comprehensive coverage across the smartphone’s mainboard. This suggests that the designed antenna array remains robust against variations in how users hold their devices. Due to its multi-element schematic and radiation diversity, the antenna system maintains stable signal transmission and reception across different handling positions. This adaptability is crucial to guarantee uninterrupted connectivity in real-world scenarios^33^.

**Fig. 11:**
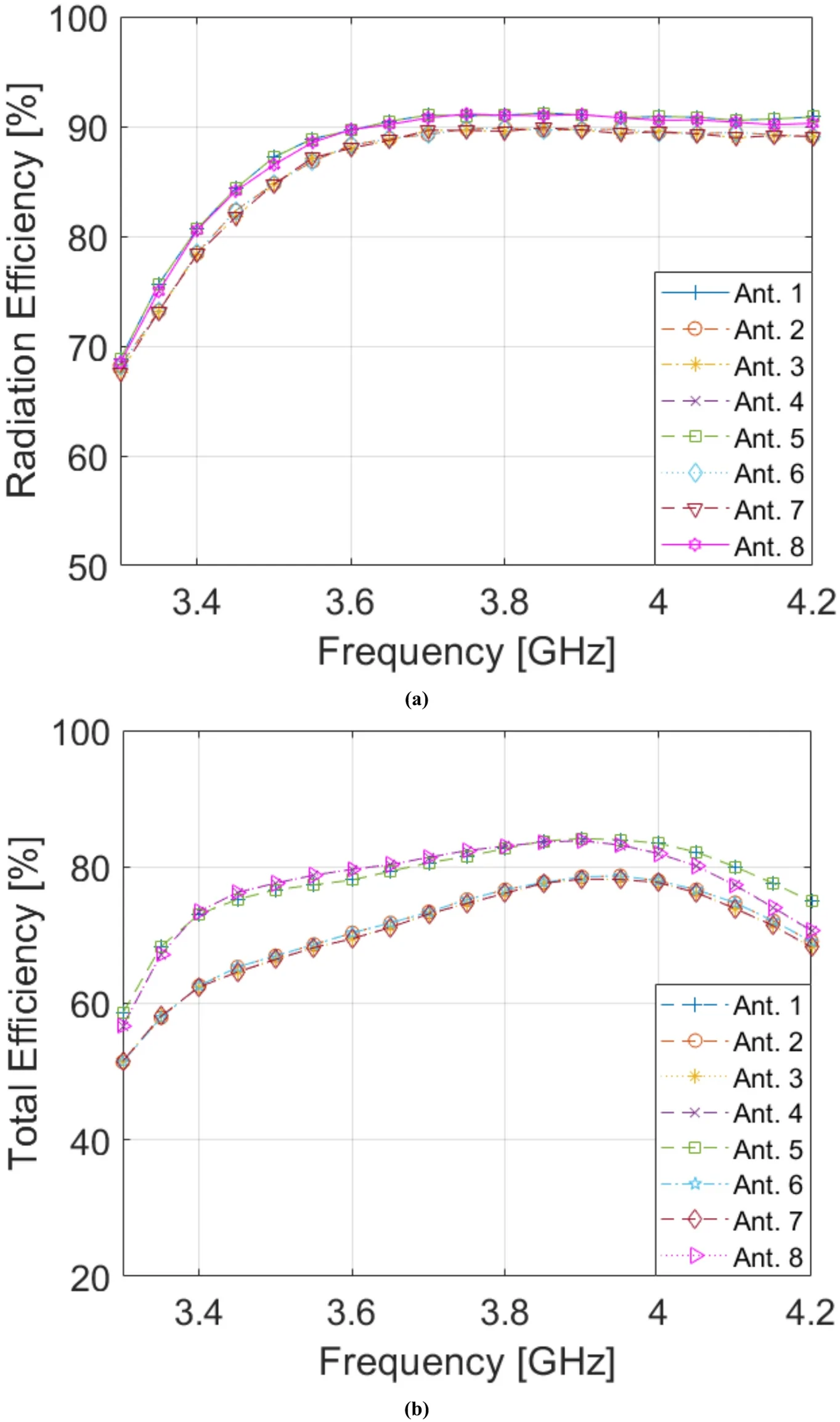
(**a**) Radiation and (**b**) total efficiency characteristics.

**Fig. 12:**
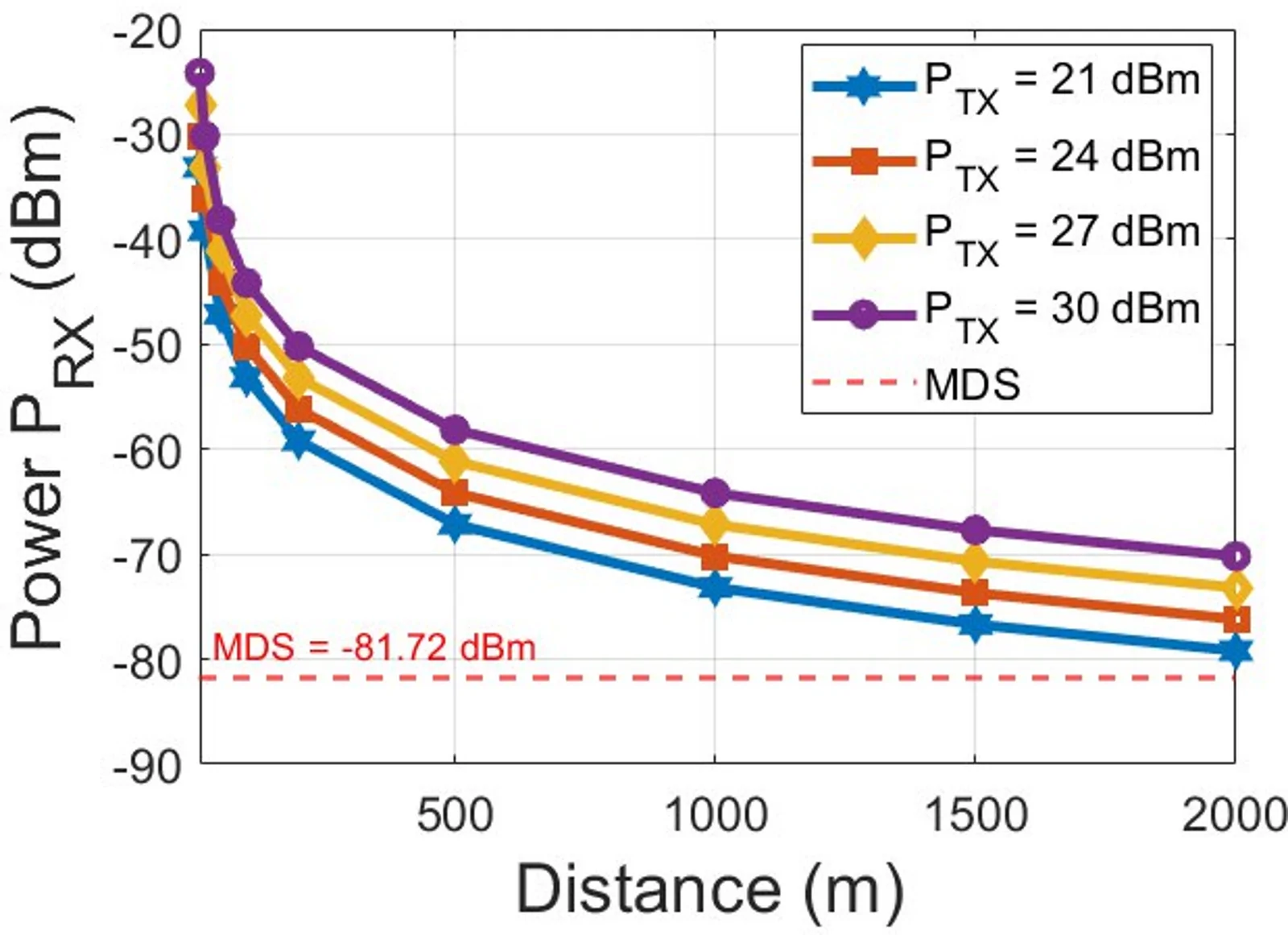
Link budget analysis for multiple transmit-power levels.

Figure 11 presents the efficiency results of the proposed system. As represented in Fig. 11 (a), each antenna element exhibits high radiation efficiency, ranging from 70% to 90%. Furthermore, total efficiency values vary between 60% and over 90%, as illustrated in Fig. 11 (b). These efficiency levels demonstrate that the antenna system performs reliably under real-world conditions, ensuring optimal functionality in practical applications, meeting the performance requirements for MIMO-enabled smartphones. High radiation efficiency indicates that a significant amount of the input power is effectively transformed into waves. Furthermore, the total efficiency considers all potential system losses, such as impedance mismatches and material absorption, offering a holistic assessment of the antenna’s real-world performance^34^. The proposed antenna employs a very compact inter-element spacing of 1.5 mm, which is significantly smaller than that of many conventional smartphone MIMO designs (as summarized in Table 3). While such close spacing typically increases mutual coupling and may affect radiation efficiency, the introduction of the T-shaped decoupling structure effectively mitigates these effects^35^. Although a slight trade-off exists due to increased near-field interaction, the achieved performance demonstrates a balanced design, with acceptable isolation levels ( ~ − 13 dB) and high radiation efficiency (approximately 70–90%). This highlights the effectiveness of the proposed approach in maintaining performance while achieving a compact form factor suitable for modern handheld devices. To evaluate the real-world communication capability of the antenna, a link budget analysis was conducted using the Friis transmission equation, which models the received power $\:{P}_{RX}$as a function of distance, transmit power $\:{P}_{TX}$, and system losses. The model accounts for: Free-space path loss (FSPL), Transmit power (P_TX_), Antenna gains at both TX and RX, Polarization mismatch loss ($\:{L}_{pol}$), and Impedance mismatch loss ($\:{L}_{imp}$). The additional mismatch losses were calculated as:

4 $$\:{L}_{pol}=-10{\mathrm{l}\mathrm{o}\mathrm{g}}_{10}\left({\mathrm{c}\mathrm{o}\mathrm{s}}^{2}\theta\:\right),{L}_{imp}=-10{\mathrm{l}\mathrm{o}\mathrm{g}}_{10}(1-\mid\:{\Gamma\:}{\mid\:}^{2})$$

where θ = 30° (typical moderate polarization mismatch), and Γ is the reflection coefficient derived from the measured S₁₁. Figure 12 presents the computed received power versus communication distance for multiple transmit power levels: 21 dBm, 24 dBm, 27 dBm, and 30 dBm. A Minimum Detectable Signal (MDS) threshold of − 81.72 dBm, based on standard receiver sensitivity at Sub 6 GHz, is indicated with a red dashed line. The plot demonstrates that reliable communication is maintained up to ~ 700 m for P_TX_ = 24 dBm and distances over 1500 m are supported for P_TX_ ≥ 27 dBm. This confirms that the antenna design provides robust link margins and long-range performance, even under realistic polarization and impedance mismatch losses, making it suitable for 5G and future Sub 6 GHz MIMO systems^35^.

## Experimental validation of the antenna

**Fig. 13:**
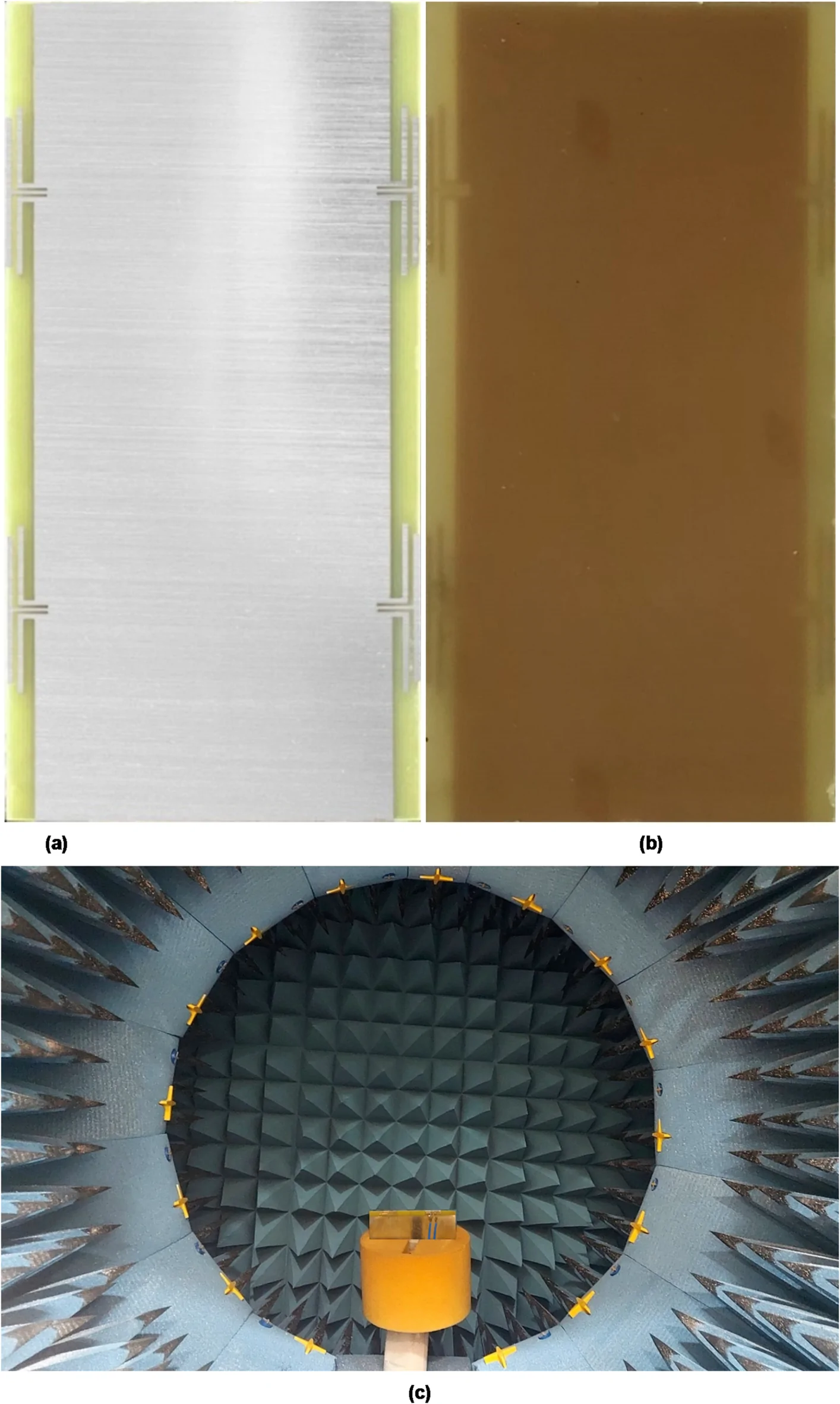
(**a**) Front-view, (**b**) back-view, (**c**) and measurement setup of the sample.

**Fig. 14:**
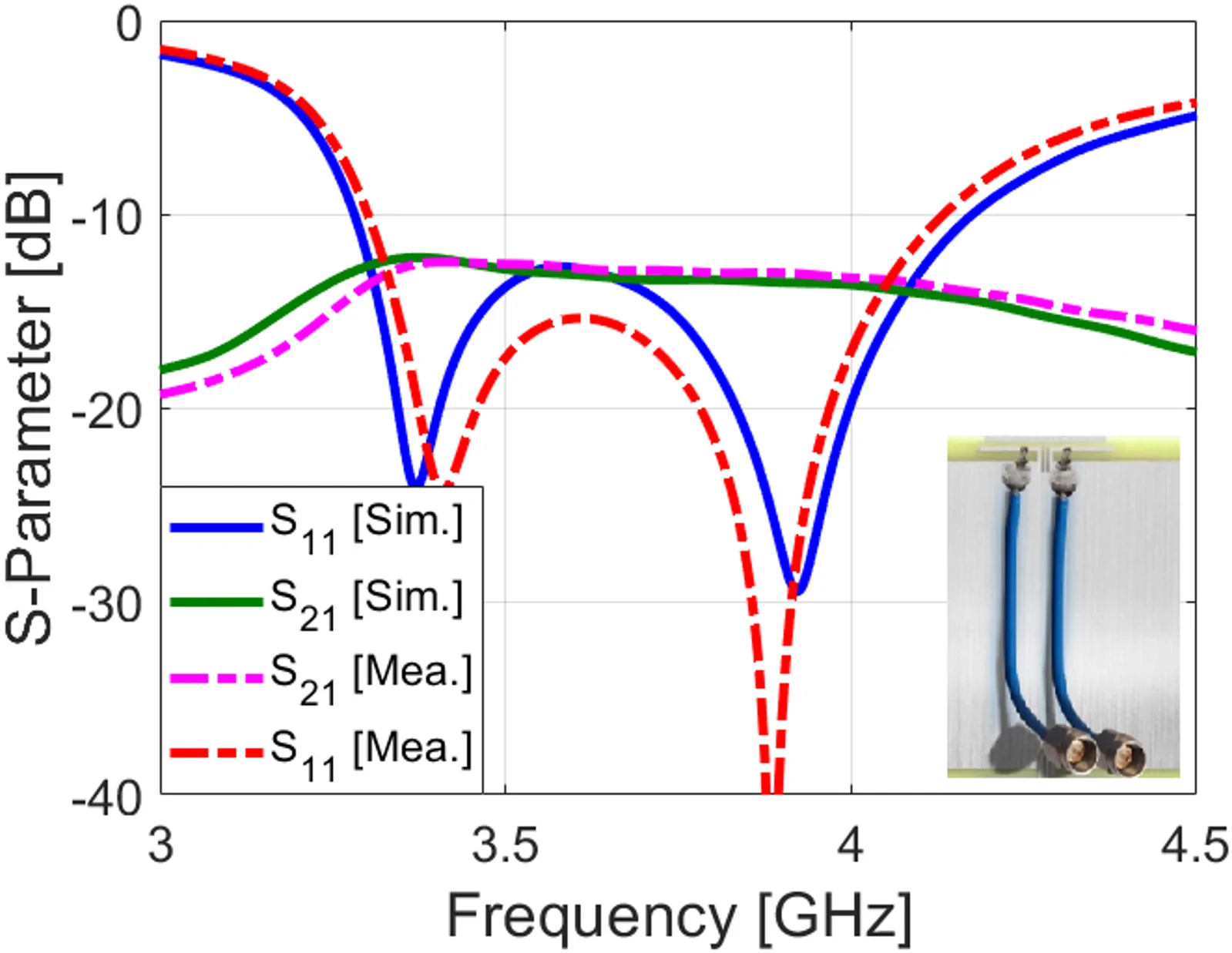
Measured and simulated S-parameters and feeding method of the antenna pair.

This section examines the key features of the fabricated prototype of the designed MIMO antenna, including its performance evaluation and comparative analysis with the simulation. Figure 13(a–b) illustrate the front and back views of the fabricated prototype. The MIMO array is implemented on a cost-effective FR4, utilizing a single-sided design with 8-element MIMO configuration. Due to the symmetrical nature of the proposed MIMO antenna array and the identical performance characteristics of its paired elements, measurements have been conducted using only a representative antenna pair, ensuring representative and efficient evaluation. In this setup, the inner conductor of a coaxial cable is securely soldered to the PIFA arms, while the outer conductor is attached to the extensive ground. Additionally, Fig. 13(c) presents the antenna’s placement inside an anechoic chamber, where comprehensive radiation measurements have been carried out to validate the system’s performance under realistic conditions. Following the fabrication and measurement process, the antenna’s performance for ports 1 and 2 is analyzed and compared with simulation results. Figure 14 provides the evaluation of the measured and simulated S-parameters, highlighting a strong correlation between the two. The measured and simulated S_11_/S_22_ values align closely, particularly in maintaining a wideband operation essential for sub-6 GHz 5G applications. As shown in the results, the antenna achieves a noteworthy bandwidth, with S_11_ values remaining below − 10 dB over the 3.25 GHz to 4.25 GHz range, confirming its suitability for broadband applications.

Moreover, the mutual coupling (S_21_) between adjacent elements remains below − 13 dB within the desired frequency band. This effectively minimizes inter-element interference, ensuring optimal isolation and independent performance of each antenna in the MIMO array. These observed characteristics emphasize the robustness of the design, making it a highly viable solution for applications requiring strong isolation, stable radiation performance, and reliable operation in electromagnetically dense environments. The ability to maintain high efficiency while minimizing interference ensures that the antenna array can effectively support high data rate transmissions.

**Fig. 15:**
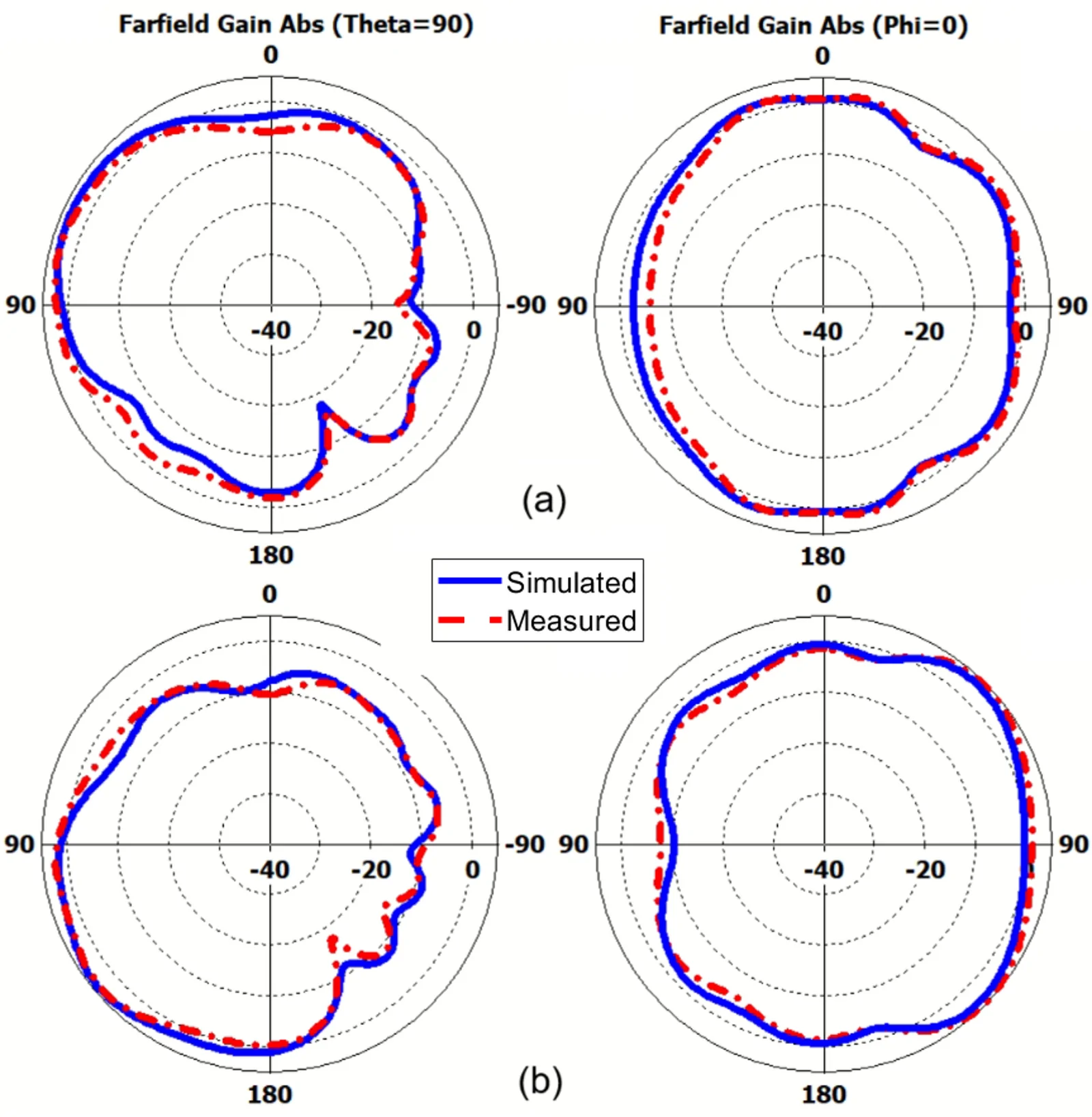
2D E-plane/H-plane radiation patterns at 3.6 GHz for (**a**) first and (**b**) second antennas under test.

**Fig. 16:**
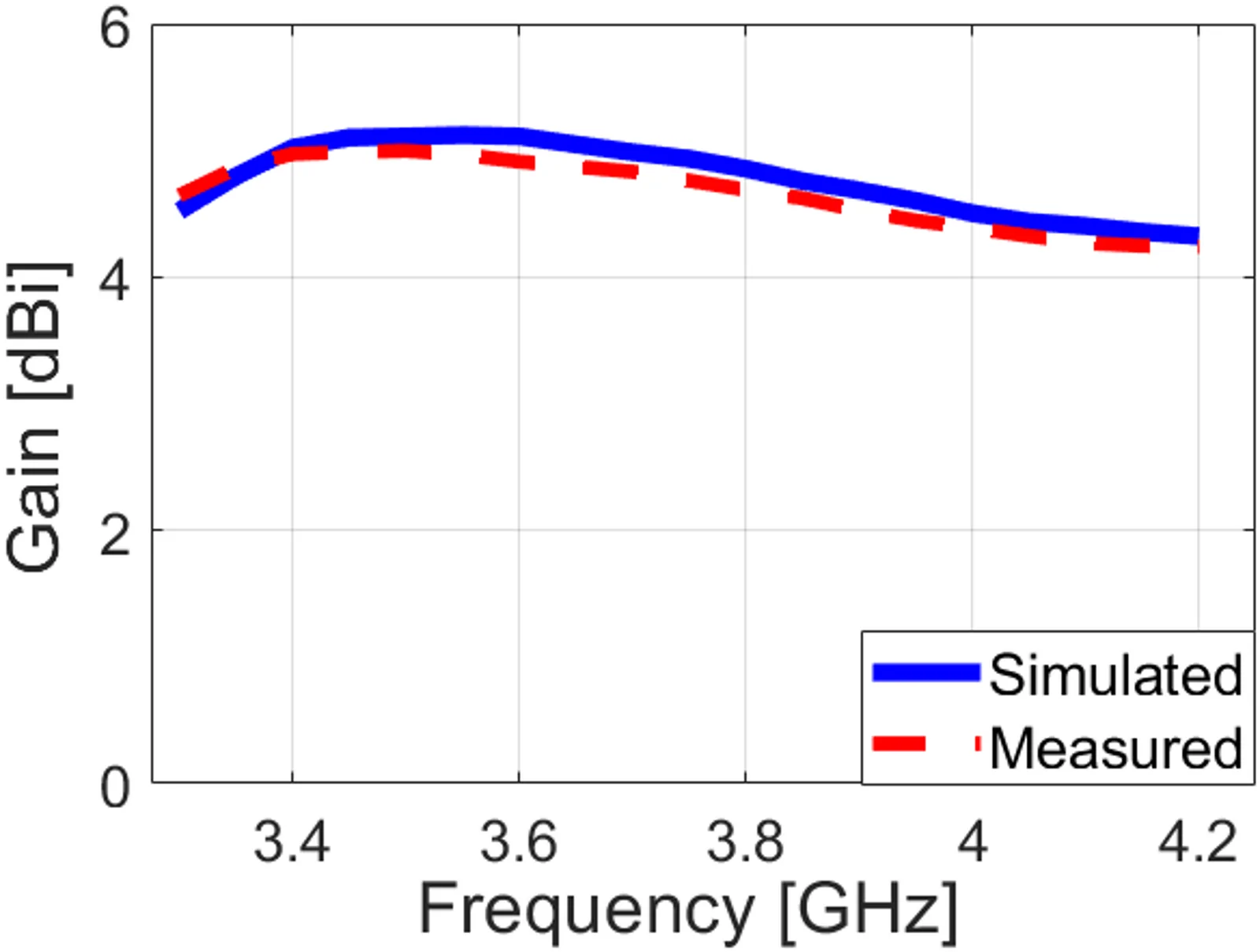
The simulated and measured gain of the antenna element.

Figure 15 illustrates the 2D radiations of the elements in both the H-plane/E-plane, obtained from both simulations and experimental measurements at 3.6 GHz. During measurements, one antenna port was actively fed, while the others were terminated with a 50 Ω load, ensuring accurate characterization and minimize external influences. The measurements indicate that the fabricated antenna delivers quasi-omnidirectional radiation patterns throughout the tested frequencies, confirming its effectiveness for scenarios requiring wide and uniform coverage. This radiation behavior ensures that the signal is distributed equally in multiple directions around the antenna element, making it well-suited for cellular applications where user orientation and device positioning can vary significantly. The quasi-omnidirectional pattern is particularly beneficial in handheld 5G devices, as it helps maintain a stable and high-quality wireless link, reducing the chances of dead zones and signal degradation due to unpredictable hand placement or movement. Additionally, the close correlation between simulated and experimental results further validates the precision of the antenna design, reinforcing its reliability for practical deployment. This strong agreement between theoretical and experimental results underscores the reliability of the investigated antenna system, ensuring that it meets the demanding performance requirements of next-generation wireless communication applications^36^. Figure 16 illustrates the gain variation of a representative antenna element, as all antenna pairs demonstrate similar radiation characteristics due to the symmetrical design. As shown, the antenna maintains a relatively stable gain with minor fluctuations between approximately 4.2 and 5 dBi across the 3.2–4.2 GHz frequency band, indicating consistent radiation performance over the operating range. This stable gain behavior further supports the suitability of the proposed antenna system for sub-6 GHz MIMO applications in handheld wireless devices.

Recent studies on compact multi-element MIMO antennas have widely employed several diversity metrics to evaluate correlation and system performance in multi-port antenna systems^37^. Therefore, several commonly used diversity metrics were analyzed in this work, including the Envelope Correlation Coefficient (ECC), Diversity Gain (DG), Mean Effective Gain (MEG), Total Active Reflection Coefficient (TARC), and Channel Capacity Loss (CCL). These parameters provide insight into the correlation between antenna elements and the overall efficiency of the MIMO system. In this work, the ECC is evaluated using the far-field radiation patterns to obtain a more accurate representation of the correlation between antenna elements. The ECC between antenna elements * i* and $\:j\:$can be calculated using the far-field radiation patterns as:

5 $$\:\begin{array}{cccc}&\:ECC={\rho\:}_{e,ij}=\frac{{\mid\:{\int\:}_{0}^{2\pi\:}{\int\:}_{0}^{\pi\:}{F}_{i}(\theta\:,\phi\:){F}_{j}^{*}(\theta\:,\phi\:)\mathrm{s}\mathrm{i}\mathrm{n}\theta\:{\hspace{0.17em}}d\theta\:d\phi\:\mid\:}^{2}}{{\int\:}_{0}^{2\pi\:}{\int\:}_{0}^{\pi\:}\mid\:{F}_{i}(\theta\:,\phi\:){\mid\:}^{2}\mathrm{s}\mathrm{i}\mathrm{n}\theta\:{\hspace{0.17em}}d\theta\:d\phi\:{\int\:}_{0}^{2\pi\:}{\int\:}_{0}^{\pi\:}\mid\:{F}_{j}(\theta\:,\phi\:){\mid\:}^{2}\mathrm{s}\mathrm{i}\mathrm{n}\theta\:{\hspace{0.17em}}d\theta\:d\phi\:}&\:&\:\end{array}$$

where $\:{F}_{i}\left(\theta\:,\phi\:\right)\:$and $\:{F}_{j}\left(\theta\:,\phi\:\right)$ represent the radiation patterns of the antenna elements. For the proposed eight-element MIMO system, the ECC values are evaluated pairwise between antenna ports, and the worst-case ECC value is used to represent the correlation performance of the antenna array. The DG is derived from the ECC and can be expressed as:

6 $$\:\begin{array}{cccc}&\:DG=10\sqrt{1-\mid\:{\rho\:}_{e,ij}{\mid\:}^{2}}&\:&\:\end{array}$$

Values close to 10 dB indicate excellent diversity performance. The MEG represents the average received power of an antenna element in a multipath propagation environment. For consistency with the radiation-pattern-based diversity analysis used in this work, the MEG of the $\:\mathrm{i}$-th antenna element is evaluated from the far-field gain patterns as

7 $$\:ME{G}_{i}=\frac{1}{8\pi\:}{\int\:}_{0}^{2\pi\:}{\int\:}_{0}^{\pi\:}\left[{G}_{\theta\:,i}(\theta\:,\phi\:)+{G}_{\phi\:,i}(\theta\:,\phi\:)\right]\mathrm{s}\mathrm{i}\mathrm{n}\theta\:{\hspace{0.17em}}d\theta\:d\phi\:$$

where $\:{G}_{\theta\:,i}(\theta\:,\phi\:)$and $\:{G}_{\phi\:,i}(\theta\:,\phi\:)$denote the gain components of the * i*-th antenna element in the $\:\theta\:$- and $\:\phi\:$-polarized directions, respectively. For good diversity performance, the difference between the MEG values of antenna elements should typically remain below − 3 dB. The TARC evaluates the active impedance behavior of the multi-port antenna system when multiple ports are excited simultaneously and can be expressed as:

8 $$\:\begin{array}{cccc}&\:TARC=\sqrt{\frac{\sum\:_{i=1}^{N}\mid\:{b}_{i}{\mid\:}^{2}}{\sum\:_{i=1}^{N}\mid\:{a}_{i}{\mid\:}^{2}}}&\:&\:\end{array}$$

where $\:{a}_{i}\:$and $\:{b}_{i}\:$represent the incident and reflected waves at the * i*-th port, respectively, and * N*denotes the number of antenna ports.

**Fig. 17:**
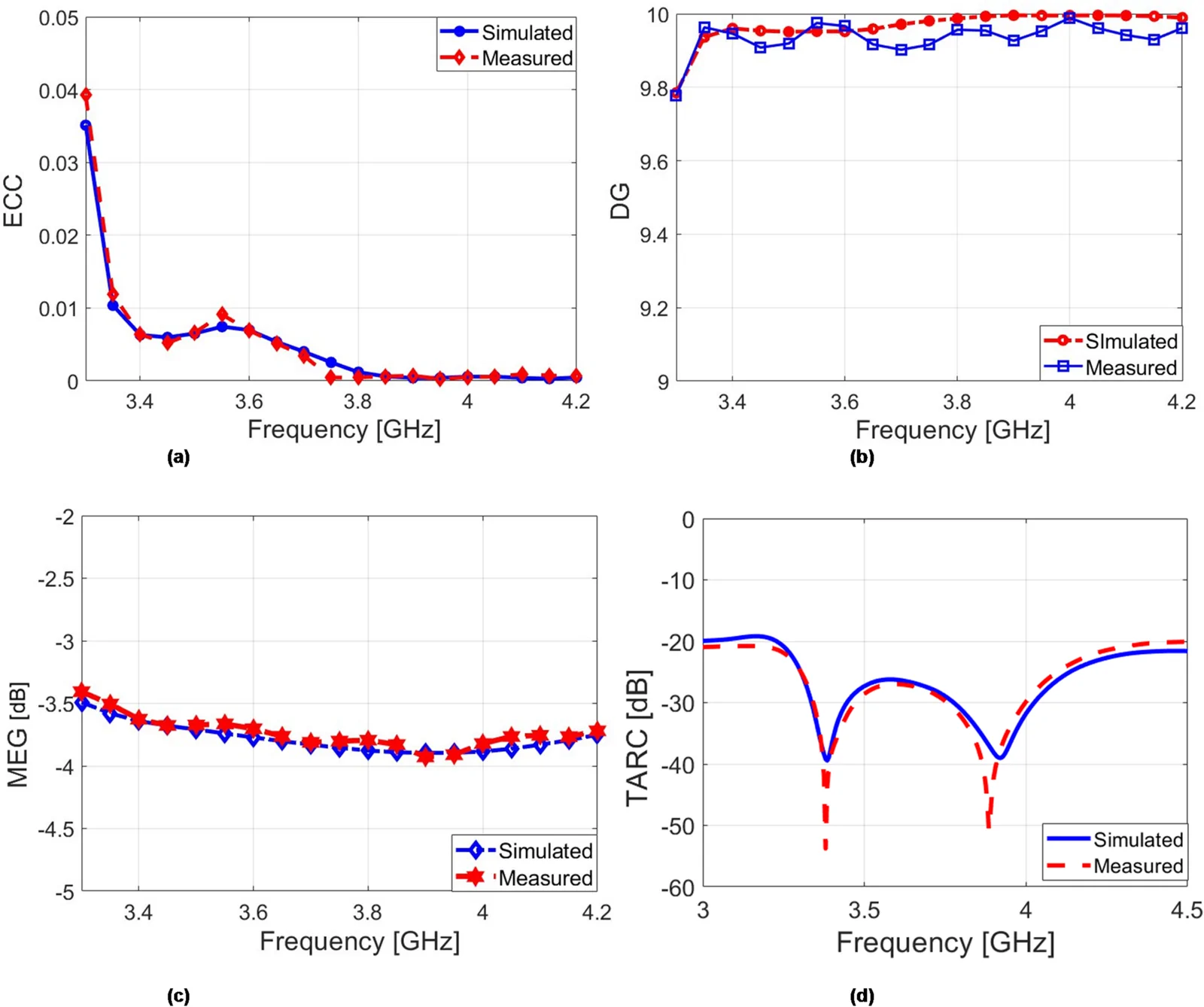
Measured and simulated comparison for (**a**) ECC, (**b**) DG, (**c**) MEG, (**d**) and TARC results.

**Fig. 18:**
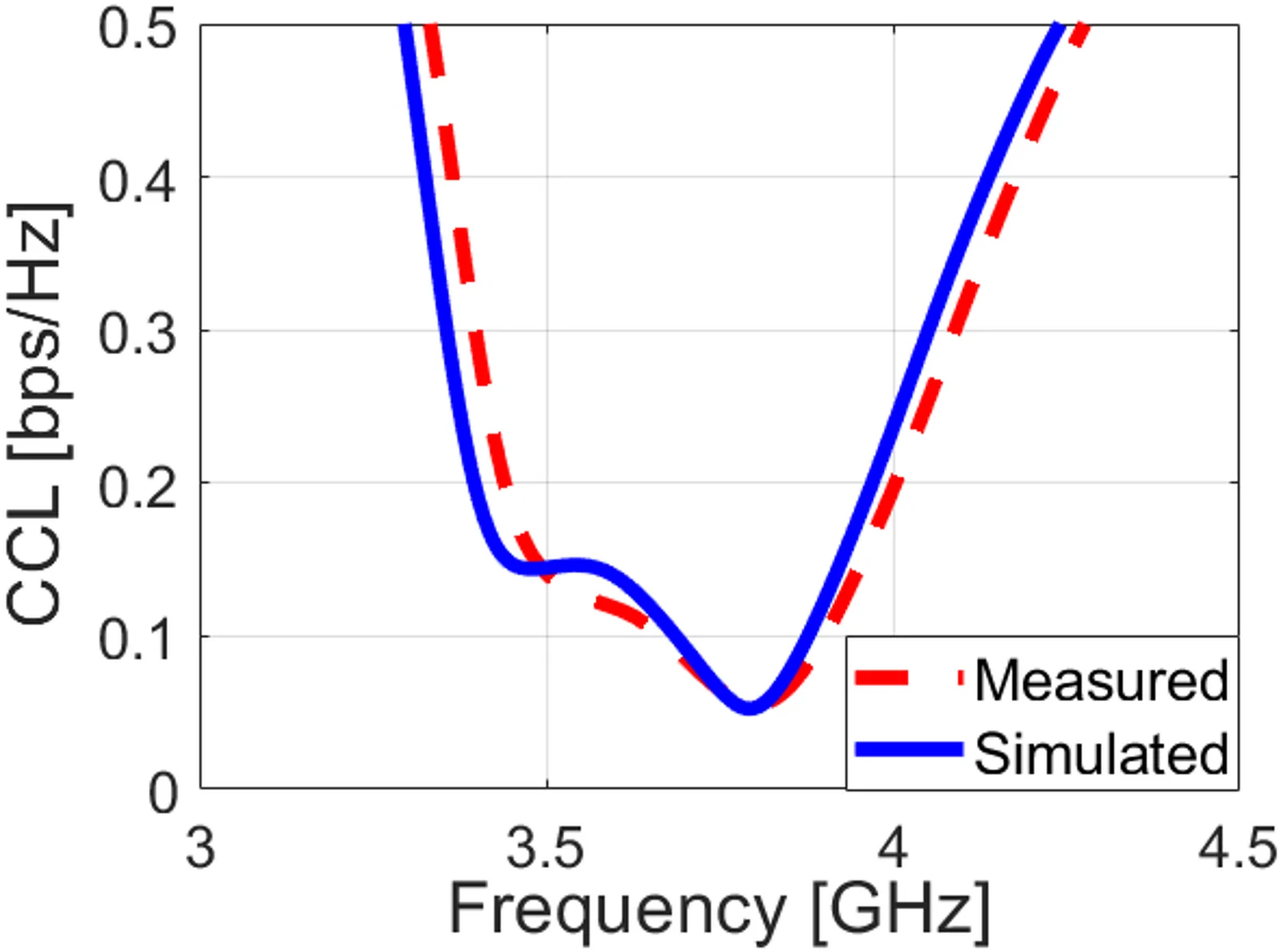
Calculated CCL function for the corresponding antenna.

Figure 17 presents the comparison between simulated and measured results of the antenna with respect to the key MIMO diversity metrics discussed above. As illustrated in Fig. 17a, the envelope correlation coefficient (ECC) remains consistently below 0.05 across the entire operating frequency range, showing strong agreement between simulated and measured results. Such low ECC values indicate minimal correlation between antenna elements, which ensures effective spatial diversity and reliable MIMO performance. The diversity gain (DG), shown in Fig. 17b, remains close to the theoretical optimum of 10 dB throughout the operating band. This confirms that the proposed antenna array provides excellent diversity characteristics and efficient signal decorrelation. Figure 17c presents the mean effective gain (MEG), which varies between approximately − 3.5 and − 4.5 dB across the frequency band. These values fall within the acceptable range for practical MIMO systems and indicate a balanced distribution of received power among the antenna elements, which is important for stable diversity performance. Finally, the total active reflection coefficient (TARC), depicted in Fig. 17d, exhibits low values across the operating band, with pronounced minima near 3.5 GHz and 4.0 GHz. These results indicate good impedance behavior and reduced reflected power when multiple antenna ports are excited simultaneously. Although slight deviations between simulated and measured responses are observed, the overall trends remain consistent, confirming the reliability of the proposed design. Overall, the combination of low ECC, near-ideal DG, stable MEG, and reduced TARC demonstrates that the proposed antenna array provides strong diversity performance and efficient power transmission, making it well suited for MIMO operation within the targeted sub-6 GHz frequency band.To further assess the MIMO performance of the proposed antenna system, the Channel Capacity Loss (CCL) was evaluated^38^. CCL quantifies the loss in the theoretical channel capacity caused by correlation and coupling between antenna elements. It is derived from the correlation matrix obtained from the S-parameters of the antenna system and can be expressed as:

9 $$\:CCL=-{\mathrm{l}\mathrm{o}\mathrm{g}}_{2}(\mathrm{d}\mathrm{e}\mathrm{t}({\Psi\:}\left)\right)$$

where $\:{\Psi\:}\:$represents the correlation matrix constructed from the S-parameters of the antenna array. For efficient MIMO performance, the CCL value should remain below 0.4 bits/s/Hz across the operating frequency band. The calculated CCL results, shown in Fig. 18, indicate that the proposed antenna maintains very low channel capacity loss, remaining well below the acceptable threshold throughout the entire operating frequency range. This confirms that the antenna array exhibits low correlation and high diversity performance, making it suitable for high-capacity MIMO communication systems^39^.

## SAR analysis and user-hand impact

**Fig. 19:**
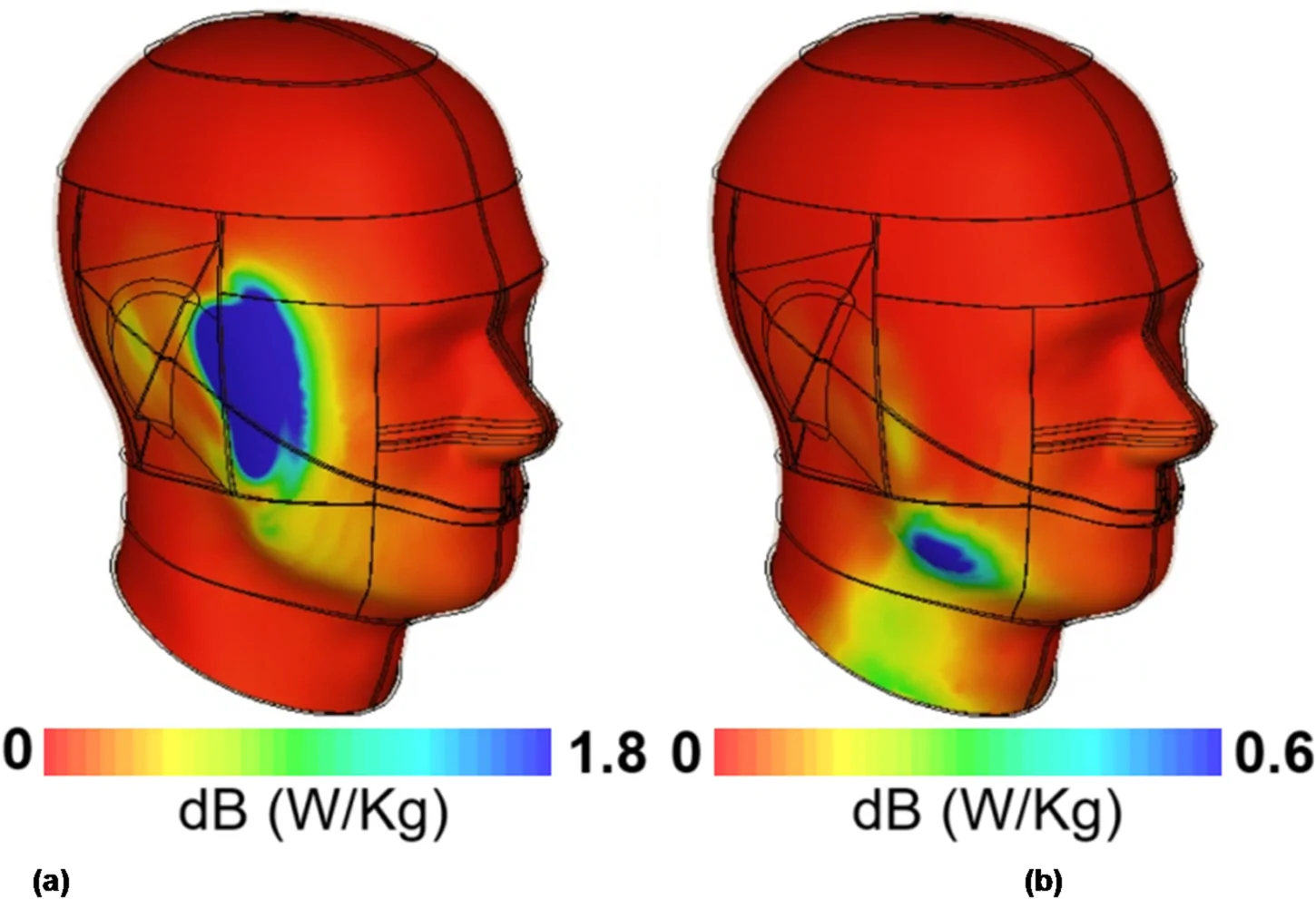
SAR results of (**a**) antenna 2 and (**b**) antenna 5.

The Specific Absorption Rate (SAR) represents the amount of radiofrequency (RF) electromagnetic energy absorbed by biological tissue per unit mass and is measured in watts per kilogram (W/kg) which is considered as a critical safety metric for handheld wireless devices and is regulated by international standards to ensure user safety^40^. In this section, the SAR evaluation was performed for the 8‑element MIMO antenna in accordance with IEEE and IEC guidelines^41^. Following standard regulatory practice, each antenna port was excited individually with 1 W input power to identify worst‑case localized exposure under controlled and repeatable conditions. This enables consistent comparison among antenna elements and ensures compliance for any single‑port excitation scenario. The SAR distribution was evaluated for 1 g of tissue, and the obtained results were compared against the 2.0 W/kg regulatory exposure limit specified by IEC/ICNIRP standards. As shown in Fig. 19, the maximum SAR value under worst‑case single‑port excitation is 1.8 W/kg, while the minimum SAR value is approximately 0.6 W/kg, both of which remain within the prescribed safety limit. These results confirm that the proposed antenna system operates safely even under worst‑case excitation conditions. The analysis indicates that Antenna 2 exhibits the highest SAR, whereas Antenna 5 demonstrates the lowest SAR among all elements. This variation is primarily attributed to the relative placement of the antenna elements within the handset layout. Specifically, Antenna 2 is positioned closest to the head phantom, resulting in higher localized absorption (Fig. 19a), while Antenna 5 is located farther from the phantom, leading to significantly reduced SAR levels (Fig. 19b). These observations highlight the strong influence of antenna placement on user exposure and emphasize the importance of spatial distribution in MIMO smartphone design. In addition to individual‑port excitation, SAR was also evaluated under a simultaneous multi‑port excitation scenario, where all eight ports were excited concurrently with equal input power summing to a total of 1 W (125 mW per port). Under this condition, the maximum observed 1 g‑SAR was approximately 1.2 W/kg, which remains well below the 2.0 W/kg regulatory threshold. This confirms that the proposed antenna system satisfies SAR safety requirements even under combined excitation conditions representative of practical MIMO operation. Overall, the SAR analysis demonstrates that the proposed antenna maintains compliance with international exposure standards across all evaluated scenarios, while preserving robust electromagnetic performance. These results confirm the suitability of the design for safe and reliable integration into next‑generation handheld devices.

**Fig. 20:**
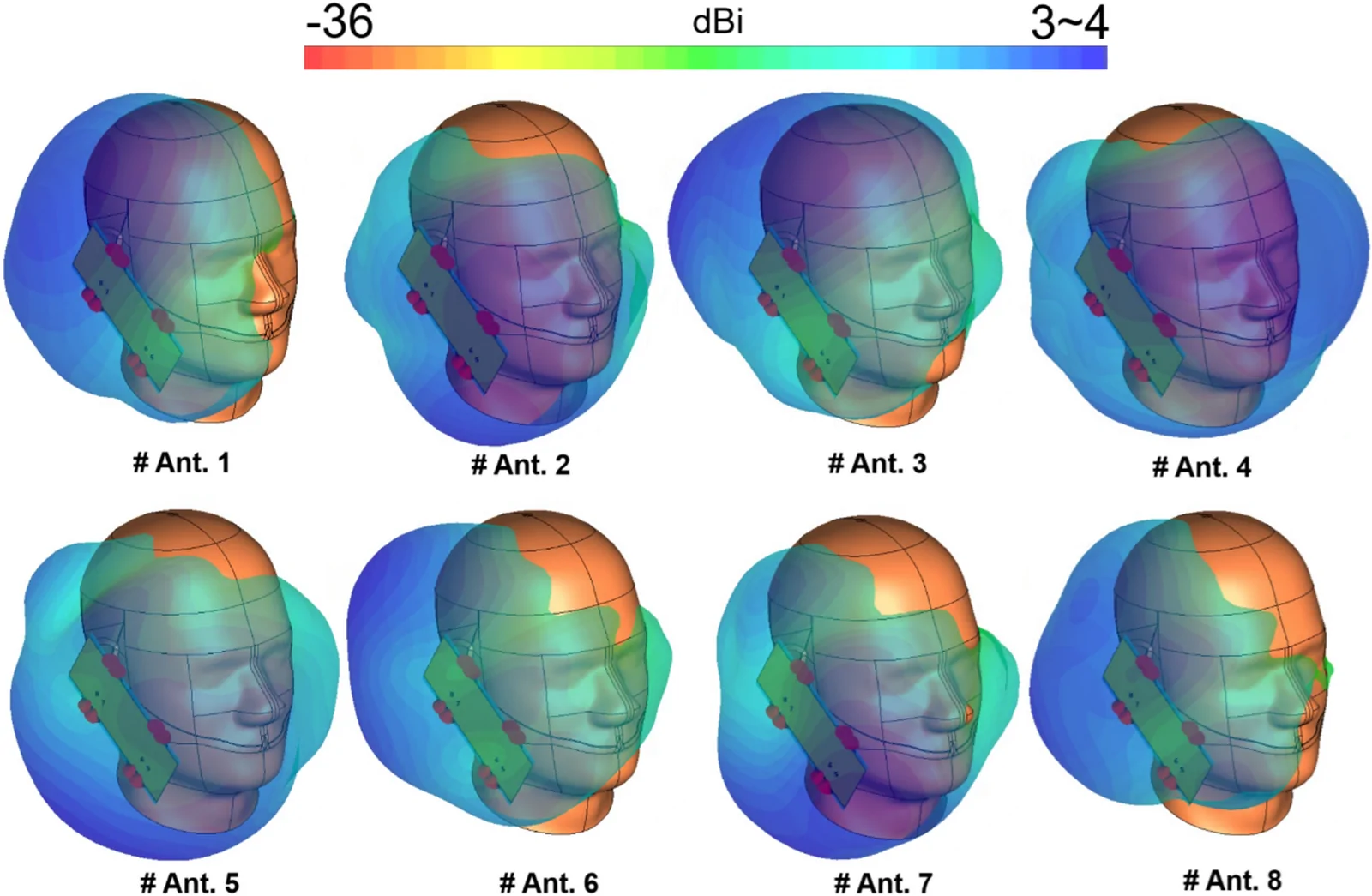
Talk-mode radiations at 3.6 GHz.

**Fig. 21:**
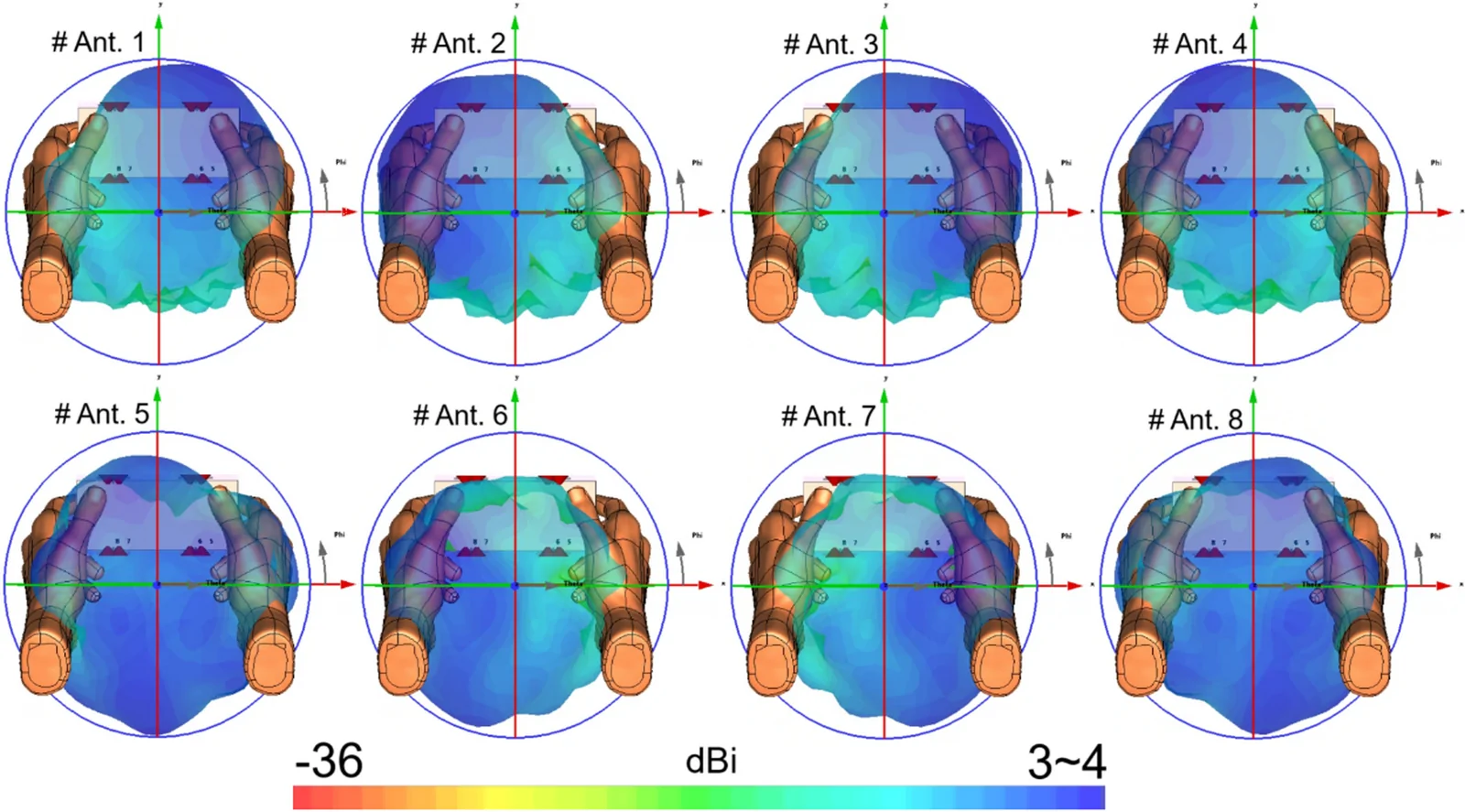
Data-mode radiations at 3.6 GHz.

**Fig. 22:**
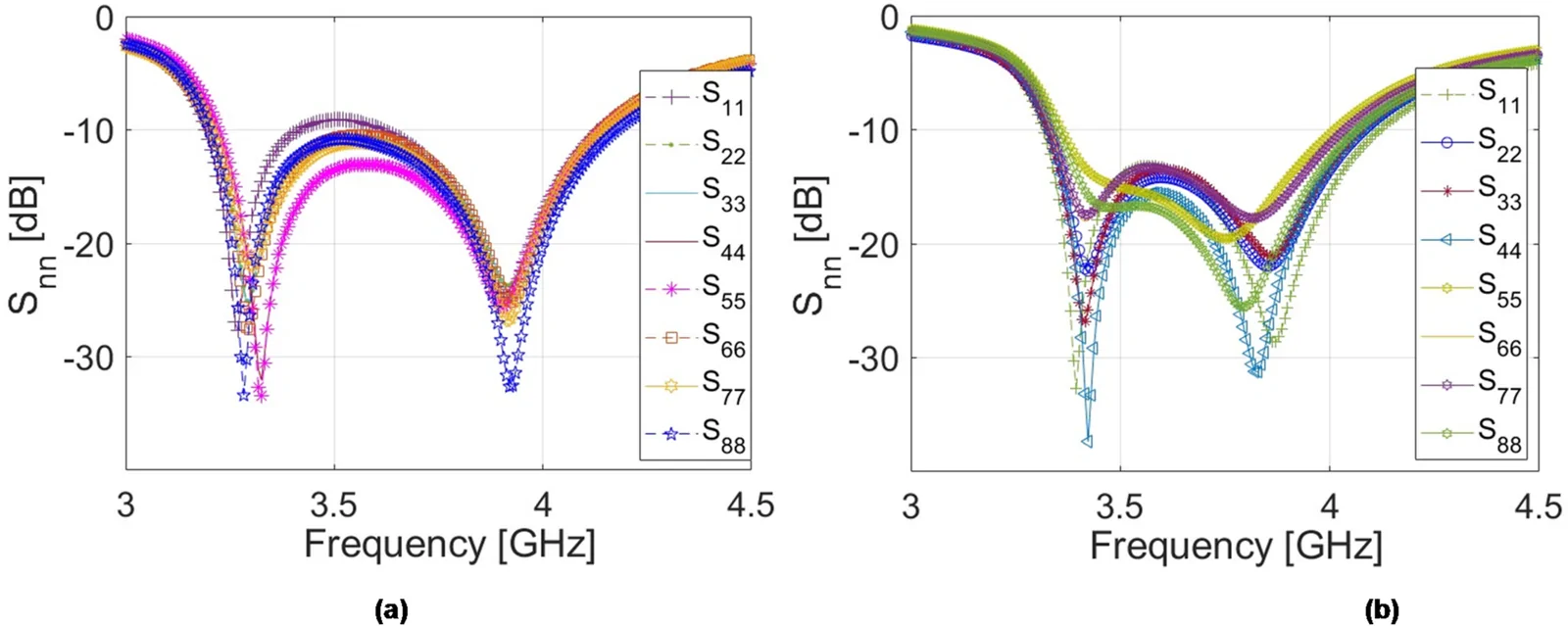
Reflection coefficient (S_nn_) results of the MIMO elements in (**a**) talk-mode and (**b**) double-hand mode.

Furthermore, the 3D radiations of the suggested MIMO design elements at 3.6 GHz have been analyzed for both talk and data modes, as illustrated in Figs. 20 and 21. Generally, user interaction, such as the presence of a hand or head, tends to degrade the radiation performance due to absorption and scattering effects. However, as shown in Figs. 20 and 21, the investigated antenna design exhibits only a slight reduction in radiation performance while still maintaining adequate gain and pattern coverage. This ensures the antenna’s suitability for cellular applications, as it retains reliable communication performance even in user-influenced scenarios^42^. To evaluate the robustness of the proposed antenna under realistic usage scenarios, the reflection coefficients (S_11_ to S_88_) were assessed for two practical user conditions: Talk-mode (where the device is held close to the head) and Double-hand mode (gripped with both hands). The results are illustrated in Fig. 22. As shown, although minor detuning and variation in reflection levels are observed due to user proximity, all antenna elements maintain satisfactory impedance matching across the operating band of 3.25–4.25 GHz. This confirms the stability and reliability of the antenna performance, supporting its suitability for smartphone applications.

##  Comparison

Table 3 presents a detailed performance comparison between the suggested antenna array and various existing designs documented in the literature^8–25^. Key parameters including antenna types, impedance bandwidth, efficiency, overall size, isolation, and ECC are evaluated. The comparison reveals that the newly developed array exhibits significant improvements, particularly in terms of broader operational bandwidth, enhanced radiation efficiency, and minimized mutual coupling. The proposed antenna system achieves a broad bandwidth exceeding 1 GHz, making it highly suitable for 5G cellular communications requiring high data rates and reduced latency^43^. A distinguishing feature of this design is its compact and planar structure, which facilitates seamless integration onto a single-layer FR4 substrate. This design approach not only simplifies fabrication but also enhances compatibility with existing smartphone architectures. Additionally, the antenna elements are strategically positioned to optimize radiation coverage across multiple sides of the device, ensuring robust signal transmission and reception regardless of the smartphone’s orientation. Furthermore, the mutual coupling function is effectively reduced due to the incorporation of modified T-shaped isolation strips, which help maintain isolation levels below − 13 dB. The low ECC values, measuring below 0.05, confirm the excellent diversity performance of the system, which is crucial for maintaining strong MIMO capabilities. Compared to other designs, the antenna array demonstrates an optimal trade-off between size, efficiency, and bandwidth, making it a promising solution for modern 5G smartphones. Overall, the findings demonstrate that the proposed MIMO antenna system not only fulfills but surpasses several key design criteria in comparison to existing solutions. Its combination of wideband operation, compact form factor, high efficiency, and strong isolation makes it well-suited for emerging wireless technologies, ensuring consistent performance in diverse and dynamic user environments. Furthermore, robust performance is maintained under realistic user scenarios and SAR compliance, and the demonstrated layout flexibility and mmWave scalability highlight the suitability of the antenna for next generation 5G and future 6G mobile platforms.

**Table 3:** Comparison table.

References	Antenna type	Bandwidth (GHz)	Efficiency (%)	Overall Size (mm^2^)	Spacing (mm)	Isolation (dB)	ECC
^8^	S-pattern monopole	3.45–3.55 (0.1)	50–68	130 × 50	31	15	< 0.30
^9^	Bent strip	3.4–3.6 (0.2)	40–70	150 × 63	16	10	< 0.1
^10^	Folded dipole	3.45–3.55 (0.1)	50–70	154 × 74	30	15	< 0.1
^11^	Inverted-F radiator	3.3–3.8 (0.5)	40–75	150 × 75	25.4	13	< 0.06
^12^	Corner-truncated patch	4.4-5 (0.6)	40–80	150 × 75	24	10	< 0.1
^13^	Slot-integrated loop	3.4–3.8 (0.4)	55–70	150 × 75	18	17	< 0.03
^14^	E-pattern slot	3.4–3.6 (0.2)	40–75	150 × 75	30	19	< 0.1
^15^	L-shaped slot	3.6–4.7 (1.1)	85–95	150 × 75	35	10	< 0.08
^16^	Patch-slot	3.5–3.7 (0.2)	50–80	150 × 75	17	16	< 0.03
^17^	Metal-rimmed strip	3.4–3.6 (0.2)	40–65	150 × 75	13	18	< 0.1
^18^	Inverted T-slot	3.4–3.6 (0.2)	40–60	150 × 75	28	16	< 0.1
^19^	T-shaped monopole	3.4–3.6 (0.2)	80–90	140 × 75	< 50	15	< 0.07
^20^	Open-ended loop	3.2–3.9 (0.7)	80–95	66 × 66	33	17	< 0.1
^21^	Coaxial-fed patch	4.4–5.0 (0.6)	20–80	150 × 80	31	15	< 0.1
^22^	Integrated monopole	4.4–5.0 (0.6)	38–52	150 × 70	-	11.5	< 0.2
^23^	Coaxial-fed module	6.4–7.1 (0.7)	40–50	150 × 70	20	13	< 0.1
^24^	I-shaped strips	3.4–3.6 (0.2)	-	150 × 75	24	20	< 0.1
^25^	Loop monopole	3.4–3.6 (0.2)	60–80	150 × 78	33	15	< 0.07
This work	Coupled PIFA	3.25–4.25 (1)	65–80	150 × 75	10	13	< 0.05

## MM-wave phased array integration; proof of concept

**Fig. 23:**
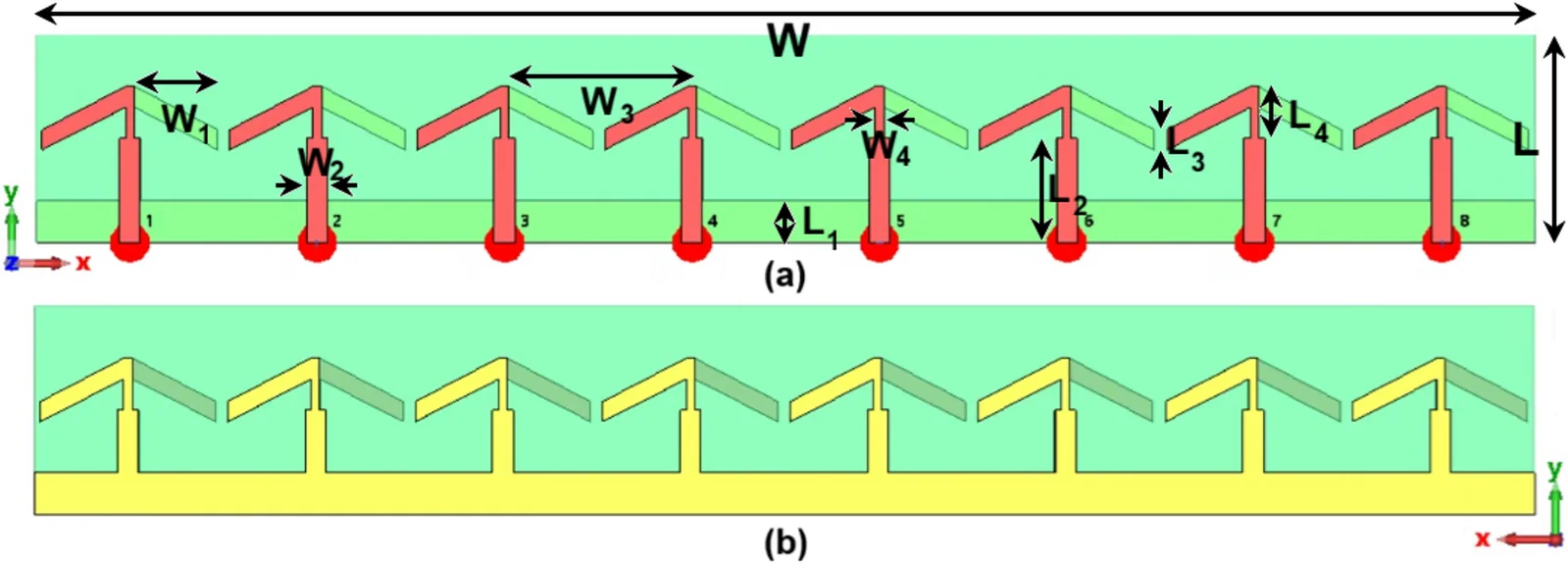
(**a**) Top-side and (**b**) bottom-side layouts of the proposed wideband mmWave phased-array.

**Fig. 24:**
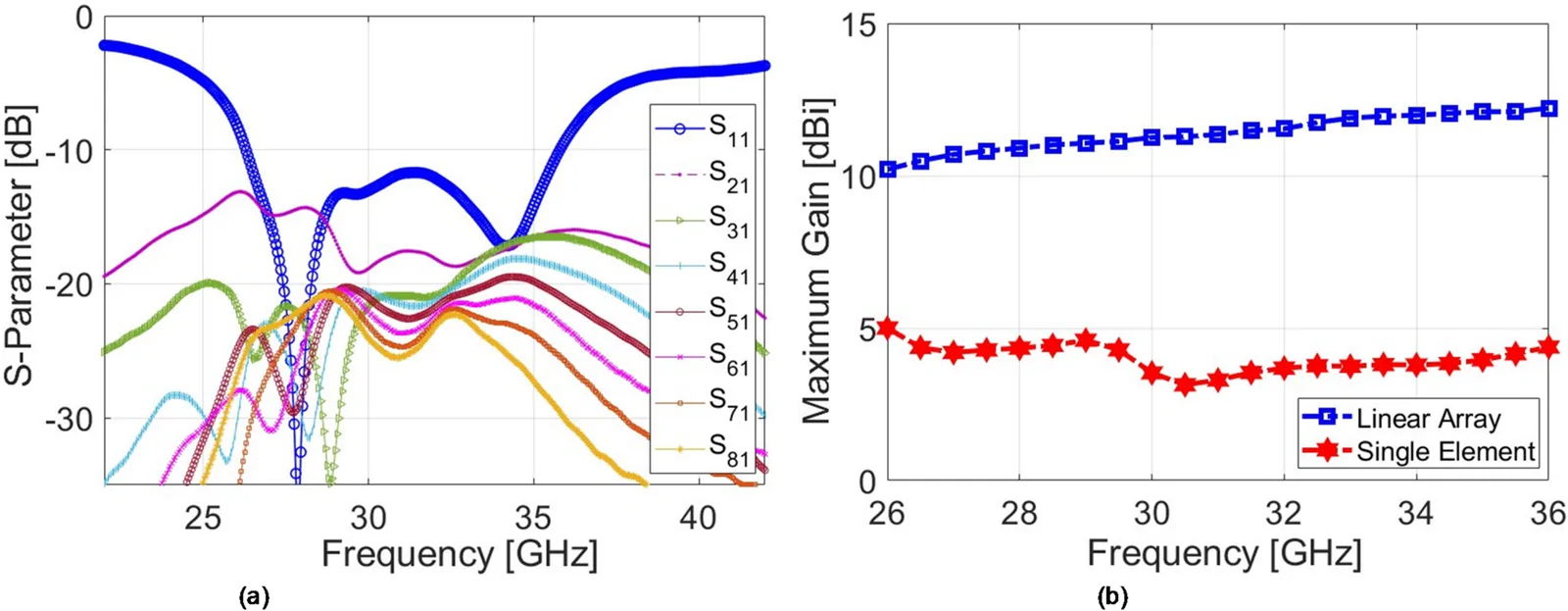
(**a**) Simulated S parameters and (**b**) gain comparison between the phased array and a single element.

Beyond the sub-6 GHz band, the incorporation of high-frequency antenna systems, particularly mmWave phased arrays, plays a critical role in extending 5G capabilities and supporting the evolution toward future 6G wireless networks^44^. To demonstrate this forward-looking capability, a compact mmWave phased-array antenna is proposed as a proof-of-concept for co-integration on a shared smartphone printed circuit board (PCB). The geometry and layout of the array are presented in Fig. 23, where eight end-fire dipole elements are arranged in a linear configuration on a compact substrate with overall dimensions of W × L. The optimized geometric parameters (in millimeters) are given as: W = 36, L = 5, W_1_ = 2, L_1_ = 1, W_2_ = 0.5, L_2_ = 2.5, W_3_ = 4.3, L_3_ = 0.5, W_4_ = 0.2, and L_4_ = 1.25. The simulated scattering parameters of the array are illustrated in Fig. 24(a), confirming a wide impedance bandwidth spanning 27–44 GHz, which effectively covers key mmWave bands, including 28, 36, 38, and 45 GHz, while maintaining acceptable inter-element coupling. A comparison between the realized gain of a single antenna element and that of the full phased array is shown in Fig. 24(b). The standalone element exhibits a gain of approximately 4–5 dBi, whereas the phased-array configuration achieves a substantial enhancement, delivering gains exceeding 12 dBi across the operational band.

**Fig. 25:**
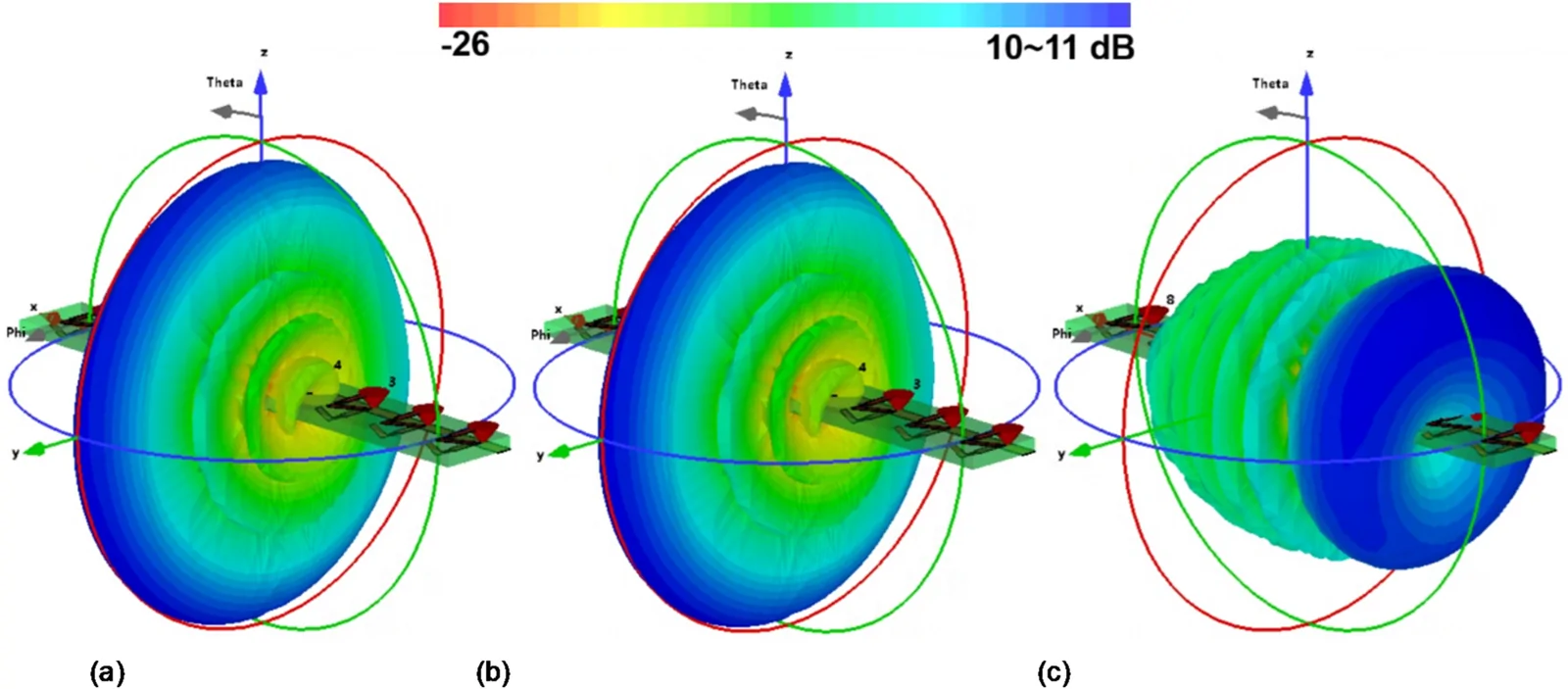
3D radiations illustrating beam steering at (**a**) 0, (**b**) 30, and (**c**) 60 degrees.

**Fig. 26:**
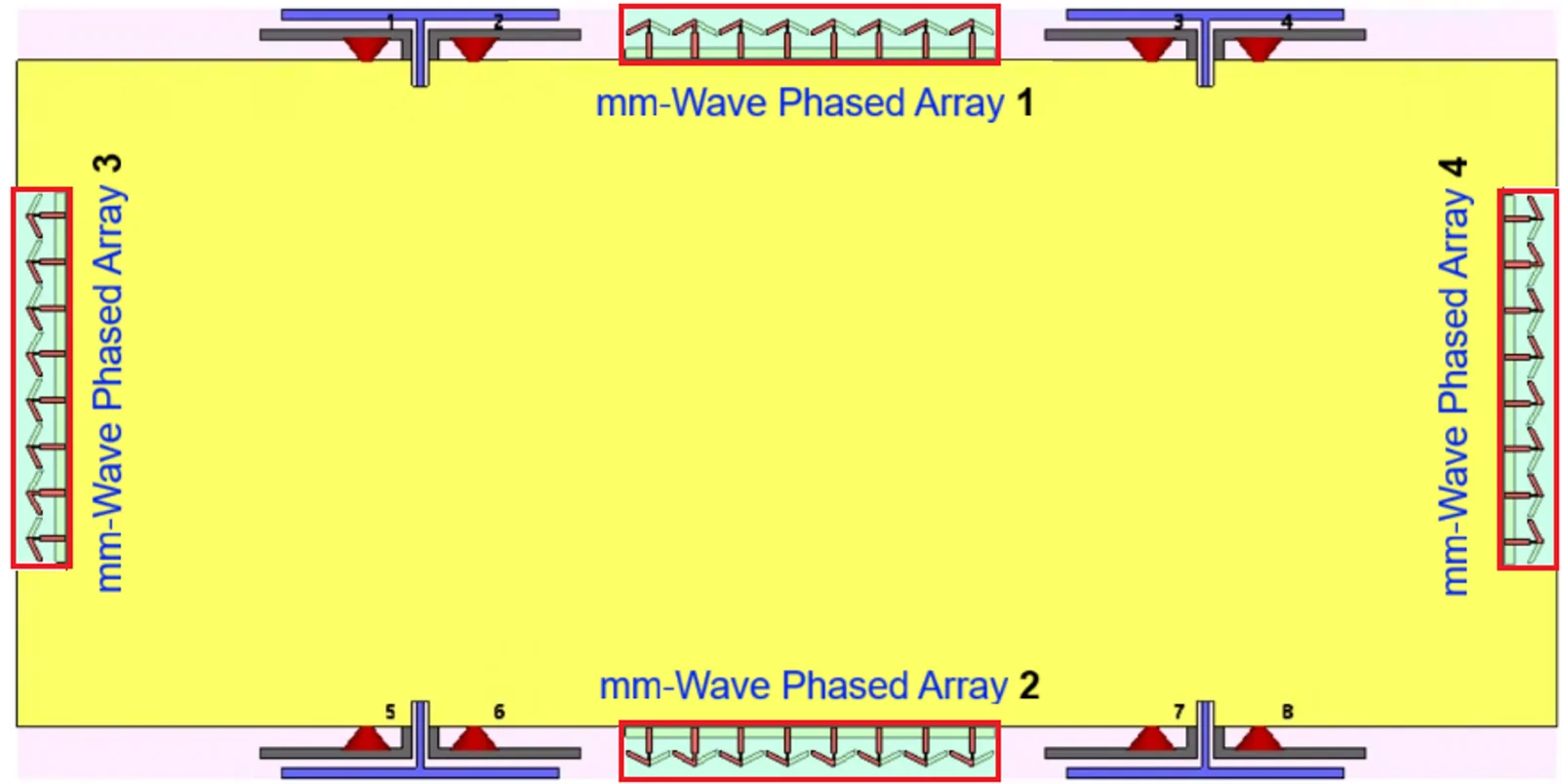
Representative integration locations of the mmWave phased array within a smartphone PCB.

The beam-steering performance of the mmWave array is demonstrated in Fig. 25, where the three-dimensional radiation patterns at 30 GHz illustrate effective beam steering at angles of 0°, 30°, and 60°, while preserving the end-fire radiation characteristics and maintaining high gain. The beam-steering capability of the proposed mmWave phased array follows the classical phased-array principle, where the radiation direction is controlled by introducing progressive phase shifts between adjacent antenna elements. The steering angle of the array can be expressed as

10 $$\:{\theta\:}_{0}={sin}^{-1}\left(\frac{\lambda\:\varDelta\:\phi\:}{2\pi\:d}\right)$$

where $\:{\theta\:}_{0}$represents the beam steering angle, $\:\lambda\:$is the wavelength, * d*is the spacing between adjacent antenna elements, and $\:\varDelta\:\phi\:$is the progressive phase shift between neighboring elements^45^. By adjusting the phase distribution across the eight dipole elements, the constructive interference direction of the radiated electromagnetic waves can be controlled, enabling the main beam to be steered toward different angular directions. The resulting radiation patterns confirm the directional beam control capability of the proposed phased-array structure, demonstrating its suitability for mmWave communication systems. Furthermore, Fig. 26 presents potential placement options of the phased array within a smartphone PCB environment. Owing to its compact form factor, the array can be accommodated in confined regions of the device, demonstrating strong layout flexibility and integration potential. Further investigations may be pursued in future work to explore the role of high-frequency antenna systems more comprehensively in next-generation 5G and 6G mobile platforms.

## Conclusion

This paper introduces an innovative antenna array system tailored for sub-6 GHz smartphone platforms, addressing the increasing need for efficient MIMO operation in 5G communication systems. The primary objective was to achieve a broad impedance bandwidth ranging from 3.25 to 4.25 GHz with suitable mutual coupling among the closely spaced elements and ensuring seamless integration with next-generation wireless communication standards. The antenna system is implemented on an FR4, a cost-effective and widely utilized material in smartphone manufacturing. Despite its affordability, the design maintains high efficiency and strong radiation performance, making it a practical solution for modern mobile devices. The antenna configuration consists of eight PIFAs with discrete feeding points, strategically positioned to optimize spatial diversity and minimize mutual coupling. Performance evaluations reveal that the developed antenna array surpasses previously reported designs in multiple critical aspects. The system exhibits significantly enhanced radiation efficiency, broader coverage, and superior isolation, with minimized ECC and TARC values. Furthermore, the planar single-layer structure eliminates ohmic losses associated with multilayer designs, thereby enhancing overall efficiency and simplifying integration into compact handheld devices. Experimental measurements closely align with simulation results, reinforcing the reliability and feasibility of the proposed design. Beyond electromagnetic performance, the work includes equivalent-circuit modeling and theoretical analysis to provide physical insight into the resonance and decoupling mechanisms, as well as a link-budget evaluation to verify system-level feasibility for 5G operation. Finally, a co-integrated mmWave phased array is introduced as a proof of concept, demonstrating the scalability of the proposed architecture toward hybrid sub-6 GHz and mmWave platforms, making it suitable for future 5G and 6G mobile devices.

## Data Availability

The corresponding author (n.ojaroudiparchin@napier.ac.uk), can be contacted to request the data from this study, subject to reasonable request.

## References

[1] Osseiran, A. et al. Scenarios for 5G mobile and wireless communications: The vision of the METIS project. *IEEE Commun. Mag.* **52** (5), 26–35 (2014).

[2] Wang, Y. et al. 5G mobile: Spectrum broadening to higher-frequency bands to support high data rates. *IEEE Veh. Technol. Mag.* **9**, 39–46 (2014).

[3] Al-Yasir, Y. et al. Green and highly efficient MIMO transceiver system for 5G heterogenous networks. *IEEE Trans. Green. Commun. Netw.* (2022).

[4] Luo, F. L. & Zhang, C. *Massive MIMO for 5G: Theory, Implementation and Prototyping, Signal Processing for 5G: Algorithms and Implementations*. 189–230 (IEEE, 2016).

[5] Sharawi, M. S. Printed MIMO Antenna Engineering. (Artech House, 2014).

[6] Zhang, K., Wang, Y., Burokur, S. N. & Wu, Q. Generating dual-polarized vortex beam by detour phase: From phase gradient metasurfaces to metagratings. *IEEE Trans. Microwave Theory Tech.* **70**, 200–209 (2022).

[7] Cao, Z., Chang, L., Li, Y., Wei, K. & Zhang, Z. Dual-band decoupling between extremely closely spaced PIFAs based on LC resonator. In *IEEE Transactions on Antennas and Propagation*. Vol. 73(11). 8391–8399 (2025).

[8] Kiani, S. H. et al. Multiple elements MIMO antenna system with broadband operation for 5th generation smart phones. *IEEE Access* **10**, 38446–38457 (2022).

[9] Ye, Y., Zhao, X. & Wang, J. Compact high-isolated MIMO antenna module with chip capacitive decoupler for 5G mobile terminals. *IEEE Antennas. Wirel. Propag. Lett.* **21** (5), 928–932 (2022).

[10] Zhang, H. H. et al. Low-SAR MIMO antenna array design using characteristic modes for 5G mobile phones. *IEEE Trans. Antennas Propag.* **70** (4), 3052–3057 (2022).

[11] Zeng, W. F., Chen, F. C. & Chu, Q. X. Bandwidth-enhanced 5G mobile phone antenna pair with tunable electric field null. In *IEEE Transactions on Antennas and Propagation*. vol. 71(2). 1960–1964 (2023).

[12] Tian, X. & Du, Z. Wideband shared-radiator four-element MIMO antenna module for 5G mobile terminals. *IEEE Trans. Antennas Propag.* **71** (6), 4799–4811 (2023).

[13] Parchin, N. O. et al. Four-element/eight-port MIMO antenna system with diversity and desirable radiation for sub 6 GHz modern 5G smartphones. *IEEE Access.* **10**, 133037–133051 (2022).

[14] Abubakar, H. S. et al. Eight-port modified E-slot MIMO antenna array with enhanced isolation for 5G mobile phone. *Electronics* **12** (2), 316 (2023).

[15] Wang, Z., You, W., Yang, M., Nie, W. & Mu, W. Design of MIMO antenna with double L-shaped structure for 5G NR. *Symmetry* **15** (3), 579 (2023).

[16] Parchin, N. O. et al. An efficient antenna system with improved radiation for multi-standard/multi-mode 5G cellular communications. *Sci. Rep.* **13**, 4179 (2023). 36914740 10.1038/s41598-023-31407-z PMC10011579

[17] Ji, Z. et al. Low mutual coupling design for 5G MIMO antennas using multi-feed technology and its application on metal-rimmed mobile phones. In *IEEE Access*. Vol. 9. 151023–151036 (2021).

[18] Fang, Y., Jia, Y., Zhu, J. Q., Liu, Y. & An, J. Self-decoupling, shared-aperture, eight-antenna MIMO array with MIMO-SAR reduction. In *IEEE Transactions on Antennas and Propagation*. Vol. 72(2). 1905–1910 (2024).

[19] Zhang, H. H. et al. Low-SAR four-antenna MIMO array for 5G mobile phones based on the theory of characteristic modes of composite PEC-lossy dielectric structures. *IEEE Trans. Antennas Propag.* **70** (3), 1623–1631 (2022).

[20] Abdelghany, M. A. et al. Compact sub-6 GHz four-element flexible antenna for 5G applications. *Electronics* **13**, 537 (2024).

[21] Cheng, B. & Du, Z. A wideband low-profile microstrip MIMO antenna for 5G mobile phones. In *IEEE Transactions on Antennas and Propagation*. Vol. 70(2). 1476–1481 (2022).

[22] Ahn, J. et al., Wideband 5G N77/N79 4 × 4 MIMO antenna featuring open and closed stubs for metal-rimmed smartphones with four slits. In *IEEE Antennas and Wireless Propagation Letters*. Vol. 22(12). 2798–2802 (2023).

[23] Wong, K. L., Wu, C. Y., Tsao, P. C., Hsu, S. W. & Li, W. Y. *Extremely Low-Profile Four-Antenna Module Covering U6 GHz Band and Its Extreme Receive Antennas-Aided MIMO Application for 6G Mobile Devices*. 10.1109/ACCESS.2025.3606909 (IEEE Access, 2025).

[24] Ghawbar, F. et al. Highly self-isolated 12-MIMO antenna elements for 5G mobile applications. *Electronics* **14**(7), 1424 (2025).

[25] Thakur, V., Jaglan, N. & Gupta, S. D. Side edge printed eight-element compact MIMO antenna array for 5G smartphone applications. *J. Electromagn. Waves Appl.* **36** (12), 1685–1701 (2022).

[26] CST Microwave Studio, ver. 2024 (CST, 2024).

[27] Zhao, A. & Ren, Z. Wideband MIMO antenna systems based on coupled-loop antenna for 5G N77/N78/N79 applications in mobile terminals. *IEEE Access.* **7**, 93761–93771 (2019).

[28] Faouri, Y. S. et al. A novel Meander Bowtie-shaped antenna with multi-resonant and rejection bands for modern 5G communications. *Electronics* **11**(5), 821 (2022).

[29] Rosaline, I. et al. Four element MIMO antenna systems with decoupling lines for high-speed 5G wireless data communication. *Int. J. Antennas Propag*. **9078929**, 13 (2022).

[30] Chen, X., Zhang, S. & Li, Q. A review of mutual coupling in MIMO systems. In *IEEE Access*. Vol. 6. 24706–24719 (2018).

[31] Kumar, A. et al. Characterization and modeling of self-diplexing MIMO antenna for endoscopy capsule applications. *Int. J. Commun. Syst.* **38**(12), e70179 (2025).

[32] Elabd, R. H. Low mutual coupling miniaturized dual-band quad-port MIMO antenna array using decoupling structure for 5G smartphones. *Discov Appl. Sci.* **6**, 189 (2024).

[33] Sufian, M. A. et al. Mutual coupling reduction of a circularly polarized MIMO antenna using parasitic elements and DGS for V2X communications. In *IEEE Access*. Vol. 10. 56388–56400 (2022).

[34] Vaughan, R. G. & Andersen, J. B. Antenna diversity in mobile communication. *IEEE Trans. Veh. Technol.* **VT-36** (4), 149–172 (1987).

[35] Chen, X., Soh, P.J. & Sharawi, M. S. MIMO antenna fundamentals and characterization methods. In *MIMO Antenna Systems for 5G and Beyond*.13–40 (IEEE, 2025).

[36] Perli, B. R. Serpent-configured quad-port MIMO antenna with dual-band operation and defected substrate–ground structure for millimeter-wave systems. *J. Infrared Millim. Terahertz Waves*. **46** (7), 43 (2025).

[37] Addepalli, T. Design of novel compact eight-element lotus-shaped UWB-MIMO antenna with triple-notch characteristics on hollow substrate. *Int. J. Commun. Syst.* **36**(8), Art. no. e5465 (2023).

[38] Addepalli, T. Effective area reduction and surface waves suppression of a novel four-element MIMO antenna exclusively designed for dual band 5G sub-6 GHz (N77/N78 and N79) applications. *Wireless Netw.* **31** (2), 1463–1479 (2025).

[39] Addepalli, T. Fractal loaded, novel, and compact two- and eight-element high diversity MIMO antenna for 5G sub-6 GHz (N77/N78 and N79) and WLAN applications, verified with TCM analysis. *Electronics* **12** (4), 952 (2023).

[40] Jamshed, M. A., Brown, T. W. C. & Héliot, F. MIMO antennas with coupling manipulation for low SAR devices. In *Low Electromagnetic Field Exposure Wireless Devices*.135–149 (Fundamentals and Recent Advances, IEEE, 2023).

[41] IEC/IEEE 62209 -528. *Measurement Procedure for the Assessment of Specific Absorption Rate of Human Exposure to Radio Frequency Fields from Hand-Held and Body-Worn Wireless Communication Devices — Part 1528: Human Models, Instrumentation, and Procedures*. (IEC & IEEE, 2020).

[42] Muhammad, A. J., Brown, T. W. C. & Héliot, F. Antenna design considerations for low SAR mobile terminals. In *Low Electromagnetic Field Exposure Wireless Devices*.115–134 (Fundamentals and Recent Advances, IEEE, 2023).

[43] Zhang, H. H. et al. Design of low-SAR mobile phone antenna: Theory and applications. *IEEE Trans. Antennas Propag.* **69** (2), 698–707 (2021).

[44] Hong, W. et al. The role of millimeter-wave technologies in 5G/6G wireless communications. *IEEE J. Microwaves* **1**(1), 101–122 (2021).

[45] Huang, H. C., Wu, J. & Cui, S. Maximally PCB-space-saving hybrid integration of millimeter-wave and microwave antennas for 5G and B5G smartphones. In *IEEE Access* **13**, 22812–22819 (2025).

